# The impact of severe perinatal events on maternity care providers: a scoping review

**DOI:** 10.1186/s12913-024-10595-y

**Published:** 2024-02-07

**Authors:** Marianne Nieuwenhuijze, Patricia Leahy-Warren, Maria Healy, Songül Aktaş, Ruveyde Aydin, Jean Calleja-Agius, Josefina Goberna-Tricas, Eleni Hadjigeorgiou, Katharina Hartmann, Lena Henriksen, Antje Horsch, Ute Lange, Margaret Murphy, Annabelle Pierron, Rainhild Schäfers, Zada Pajalic, Corine Verhoeven, Dolores Ruiz Berdun, Sheima Hossain

**Affiliations:** 1 CAPHRI, Maastricht University, Universiteitssingel 60, 6229 ER Maastricht, The Netherlands; 2 University College Cork, Cork, Ireland; 3 Queen’s University Belfast, Belfast, UK; 4 Karadeniz Technical University, Trabzon, Turkey; 5 Ondokuz Mayıs University, Samsun, Turkey; 6 University of Malta, Msida, Malta; 7 University of Barcelona, Barcelona, Spain; 8 Cyprus University of Technology, Limassol, Cyprus; 9 Mother Hood E.V, Bonn, Germany; 10 Oslo Metropolitan University, Oslo, Norway; 11 University of Lausanne, Lausanne, Switzerland; 12 University of Applied Sciences Bochum, Bochum, Germany; 13 University of Bordeaux, Bordeaux, France; 14 University of Münster, Münster, Germany; 15 VID Specialized University, Oslo, Norway; 16 Amsterdam University Medical Centres, Amsterdam, The Netherlands; 17 University of Alcalá, Madrid, Spain

**Keywords:** Maternity care, Care providers, Midwives, Obstetricians, Nurses, Severe event, Adverse event, Perinatal period, Impact, Support, Trauma

## Abstract

**Background:**

Severe events during the perinatal period can be experienced as traumatic by pregnant women, their partners or others who are closely involved. This includes maternity care providers who can be affected by being involved in or observing these events. This may have an impact on their personal well-being and professional practice, influencing quality of care. The aim of this study is to map research investigating the impact of severe events during the perinatal period on maternity care providers, and how these experiences affect their well-being and professional practice.

**Method:**

A scoping review following the manual of the Joanna Briggs Institute was undertaken. The electronic bibliographic databases included PubMed/MEDLINE, CINAHL, PsycINFO, PsycARTICLES, SocINDEX, Cochrane, Scopus, Web of Science and databases for grey literature. Records passing the two-stage screening process were assessed, and their reference lists hand searched. We included primary research papers that presented data from maternity care professionals on the impact of severe perinatal traumatic events. A descriptive content analysis and synthesis was undertaken.

**Results:**

Following a detailed systematic search and screening of 1,611 records, 57 papers were included in the scoping review. Results of the analysis identified four categories, which highlighted the impact of traumatic perinatal events on maternity care providers, mainly midwives, obstetricians and nurses: *Traumatic events*, *Impact of traumatic events on care providers*, *Changes in care providers’ practice* and *Support for care providers;* each including several subcategories.

**Conclusion:**

The impact of traumatic perinatal events on maternity care providers ranged from severe negative responses where care providers moved position or resigned from their employment in maternity care, to responses where they felt they became a better clinician. However, a substantial number appeared to be negatively affected by traumatic events without getting adequate support. Given the shortage of maternity staff and the importance of a sustainable workforce for effective maternity care, the impact of traumatic perinatal events requires serious consideration in maintaining their wellbeing and positive engagement when conducting their profession. Future research should explore which maternity care providers are mostly at risk for the impact of traumatic events and which interventions can contribute to prevention.

**Supplementary Information:**

The online version contains supplementary material available at 10.1186/s12913-024-10595-y.

## Introduction

Severe negative events can often have significant sequela on people who are directly affected or who are observers. The effects of trauma on the mental health of individuals, families and populations are well documented [1, 2]. Traumatic events can be collective events involving larger groups of people, such as an earthquake, or more individual events, such as a car accident or the loss of a loved one. Individual traumatic events can also be experienced by those receiving, observing or providing healthcare [3, 4].

Even though pregnancy and childbirth are usually positive experiences, certain events during the perinatal period can be experienced as traumatic by pregnant women/persons, their partners or others closely involved. These events range from severe complications during childbirth to expressions of disrespectful interaction between care providers [3, 4]. Acknowledging that traumatic events can happen throughout the perinatal period, most research so far has focused on childbirth. A recent woman-centred definition, developed through consultation of maternity care experts and consumer groups, describes this as: *“A traumatic childbirth experience refers to a woman’s experience of interactions and/or events directly related to childbirth that caused overwhelming distressing emotions and reactions; leading to short and/or long-term negative impacts on a woman’s health and wellbeing.”* [5]. This definition acknowledges that low-quality interactions with care providers and adverse events during childbirth can lead to birth trauma. As a consequence, women and their families experience intense emotions after a traumatic experience, which may have short- and long-term effects [6, 7].

In addition, maternity care providers, such as obstetricians, midwives, nurses, and support workers are also exposed to traumatic perinatal events and might be affected either indirectly by witnessing these events or hearing them from the women or partners involved [4, 8]. This could be considered secondary traumatic stress disorder symptoms [9, 10].

Previous research has reported a wide range of prevalence rates (12.6% to 96.9%) among maternity care providers witnessing or being involved in a traumatic perinatal event [8, 11–13]. This variability seems mostly related to methodological issues, such as the authors’ choice of definition of traumatic childbirth and eligibility criteria for participation.

Studies published in the last decade indicate that maternity care providers can be severely affected by being involved or witnessing a traumatic event during the perinatal period [12–18]. A systematic review by Andre et al. [18], reported that care providers experienced emotional distress after caring for women who had a perinatal death, including feeling stress, shock, anxiety, fear, guilt, self-blame, denial, depression, withdrawal, and fear of litigations. Wahlberg et al. [19] found that obstetricians and midwives reported intense feelings of fear, helplessness or panic in connection with a severe traumatic event. Furthermore, several studies showed that maternity care providers who experienced traumatic maternity events reported compassion fatigue, burnout, post-traumatic stress disorder, and their professional quality of life was negatively affected [9, 11, 14, 15, 20, 21].

Of note, maternity care providers who were involved in traumatic events and experienced intense adverse emotions often did not receive adequate support, either formally or informally [16, 18, 22]. In addition, some wanted to leave the profession or change their area of work to a department where there was less risk of experiencing traumatic events [17, 19, 23–25].

To improve the quality of perinatal care for women and the well-being of maternity care providers, it is important to map the literature to identify the size and scope of research evidence on this topic. The aim of this scoping review is to map research on the impact of traumatic events during the perinatal period on maternity care providers and how these experiences affect their well-being and professional practice.

## Methods

This review was carried out following the six-stage framework for scoping reviews developed by Arksey and O’Malley [26], refined by Levac et al. and the Joanna Briggs Institute [27, 28]. The Preferred Reporting Items for Systematic Reviews and Meta-analysis Protocols Extension for Scoping Reviews (PRISMA-ScR) was used as a checklist for the process [29].

This scoping review was conducted by a COST Action (CA18211) research team based in 12 countries across Europe, including backgrounds in midwifery, obstetrics, nursing, psychology, and service users.

### Identifying the research question

The research questions guiding the review were: What is known about the impact of perinatal traumatic events on maternity care providers and how do these experiences affect their professional practice and personal well-being?

### Searching for relevant papers

We started this study with a preliminary search looking for previous scoping reviews on the topic. We found none that included a broad range of traumatic events and all maternity care providers. After seeking advice from a university librarian, a systematic search of the databases PubMed/MEDLINE, CINAHL, PsycINFO, PsycARTICLES, SocINDEX, Cochrane, Scopus, Web of Science, was undertaken in June 2022 and updated in May 2023; grey literature was searched using BVS, Dialnet, EThOS and SciELO. We searched for publications using the key concepts ‘maternity care providers’, ‘impact of severe event’ and ‘maternity care’ and their synonyms (Supplement 1). The most appropriate MeSH, DeCS, keywords and terms for the search strategy were identified and adapted to each database specification as a collaborative effort within the research team. The PCC (Population (or participants)/Concept/Context) was used to organise the search terms [28] (Supplement 1).

### Study selection

We included all primary research papers with qualitative, quantitative and mixed research designs published from January 2010—May 2023, where data results pertinent to perinatal traumatic events could be extracted from maternity care professionals (midwives, doctors, nurses, etc.) providing care in hospital and community settings. We limited the selection to publications from 2010 onwards as we wanted to map the scope in recent research papers. All languages were accepted. We excluded: conferences papers; theses/dissertations; discussion papers and books; studies that reported on students; participants other than maternity care providers and studies that had perineal injuries or non-perinatal trauma as the central topic. The reference lists of systematic reviews identified were checked for any additional research papers.

The two researchers (SH, DR) who performed the search, did a first screening for eligibility of potential papers based on title and abstract. Subsequently, teams of two researchers were formed, based on their language and area of expertise. Each team was assigned a number of papers which they independently reviewed and met to agree on the final selection. In case of no agreement, the studies were evaluated by a third reviewer (MN). Extra attention was given to identifying papers through three experts, reference lists of all included papers and nine systematic reviews, as new terminology describing traumatic events during the perinatal period emerged overtime.

### Charting the data

Each researcher undertook data extraction as per the outline below:

- First author, year, title, country
- Study design and data collection method
- Study aim
- Study participants
- Measure of traumatic event
- Measure of outcomes

### Data analysis

Consistent with scoping review methodology, a descriptive content analysis was employed [28] and the data were organised into four primary categories guided by the aim of the scoping review: (1) traumatic events, (2) impact of traumatic events on care providers, (3) changes in care providers’ practice and (4) support for care providers. Furthermore, a numerical analysis of the extent, nature and distribution of the studies included in the review are also presented. A quality assessment of the included papers was not undertaken in keeping with scoping review principles [27, 30].

## Results

**Fig. 1:**
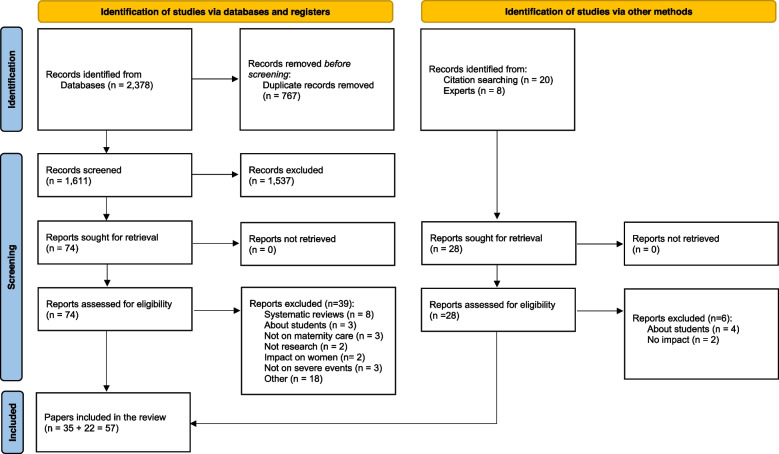
PRISMA flowchart of this study [31]

The searches in the databases identified 2,378 papers with 767 duplicates that were removed, leaving 1,611 potential papers. Subsequently, titles and abstracts from these papers were screened, excluding 1,537 papers that did not fit the scope of this review. The full-texts of the remaining 74 papers were obtained and sent to co-authors for review. Finally, 35 papers were selected that met the inclusion criteria (Fig. 1). Another 22 papers were identified through experts (*n* = 7), reference lists of the included papers (*n* = 4) and other systematic reviews related to the topic (*n* = 11).

In total, 57 papers were included and all were published in English (Supplement 2). Given the large number of papers included in this review, each paper in Supplement 2 was allocated a number corresponding to the numbers used in the results section of this paper.

### Characteristics of the studies

Studies in the included papers were conducted in 19 countries, including the United Kingdom (*n* = 9), Sweden (n = 5), Netherlands (*n* = 3), Denmark (*n* = 3), Ireland (*n* = 2), Switzerland (*n* = 2), Belgium (*n* = 1), Greece (*n* = 1) and Spain (*n* = 1). Others were from USA (*n* = 10), Australia (*n* = 8), Israel (*n* = 3), Turkey (*n* = 2), Uganda (*n* = 2), Japan (*n* = 2), New Zealand (*n* = 2), Tanzania (*n* = 1), South Africa (*n* = 1) and Canada (*n* = 1). For two papers, data were collected in two countries (16,38). There were different study designs, 20 used qualitative methods, 25 were cross-sectional surveys, one was a longitudinal survey, and 11 used a mixed-methods approach. There were a number of papers included in this review that emanated from one study. For example, for six studies, two papers were published from each study (3, 5, 28, 29, 31, 32, 34, 35, 52, 53, 54, 55) and for two studies, three papers were published from one study (42-44, 45-47). Each paper had a specific aim or research question that was the focus of the individual manuscript, which are delineated in supplement 3. Therefore, each paper was handled as a separate entity within the review.

Participants represented different professional disciplines, with midwives in the majority. Nine studies included two or more types of maternity care providers, the other papers were either focused on midwives, nurse-midwives, obstetricians, nurses or other staff. In total, 6314 midwives participated in 38 studies, 1261 nurse-midwives participated in 5 studies, 3284 obstetricians/physicians participated in 18 studies, 1598 nurses participated in 13 studies, and 67 other staff (assistant nurses and household staff) participated in 3 studies. Supplement 3 gives a full overview of the characteristics of the included papers.

As a result of the analysis, four categories were identified: *Traumatic events*, *Impact of traumatic events on care providers*, *Changes in practice* and *Support for care providers,* each including several subcategories as delineated in Table 1.

**Table 1:** Categories and subcategories

**Category 1: Traumatic events**
°*Complications and interventions during childbirth*
°*Disrespectful interactions in perinatal care*
°*Conflicts between professional philosophies*
°*Work environment*
**Category 2: Impact of traumatic events on care providers**
°*Distressing emotions and physical symptoms*
°*Impact on both personal and professional well-being*
**Category 3: Changes in care providers’ practice**
°*Changes in the way care was provided*
°*Changes in their interactions with women*
°*Changes to professional life*
**Category 4: Support for care providers**
°*Formal support*
°*Informal support*

### Traumatic events

Different approaches towards defining a traumatic event were used throughout the studies. In some studies, the researchers labelled a specific incident as traumatic (e.g., perinatal death (8) or maternal death (12)), while others gave a general description like *“events for which the respondents ‘did not feel adequately prepared’ or that they ‘found upsetting or overwhelming’”* (1). Other studies asked responders to indicate the event they found traumatic, *“leaving it in the eye of the beholder”* (3, 29), or asked participants for ‘work-related adverse or serious’ events and labelled these as traumatic when a certain number of participants scored high on a validated traumatic stress scale (2).

The term “second victim” was used to describe the traumatisation of maternity care providers who were involved in a severe event that primarily affected the mother or baby (first victim) (44, 54, 55).

We summarised the traumatic events into four subcategories: *complications and interventions during childbirth*, *disrespectful interactions in perinatal care*, *conflicts between professional philosophies*, and *work environment*.

#### Complications and interventions during childbirth

One of the most traumatising experiences reported by maternity care providers was the actual or threat of death or injury to the woman and/or child (2-4, 7-16, 18, 20-22, 25-37, 39, 41-45, 48, 50, 52-57). Experiencing unexpected, unpredictable, and uncontrollable events like shoulder dystocia, post-partum haemorrhage (PPH), a vaginal operative birth, resuscitation, overly forceful interventions, emergency births, and life-endangering situations in general, were reported as having a traumatic impact (3, 4, 9-11, 13, 18, 31, 32, 35, 36, 41, 46-48, 51). A few studies with midwives and obstetricians identified missing a diagnosis or doubting a medical decision as stressors with a severe impact (2, 26, 48).

**Table:** 

[In the case of an infant with hydrops, who was not expected to live] “As the baby’s head delivered the OB put pressure on the head to deliver the shoulder and nothing happened. We went through all the various manoeuvers but the head made no movement past the neck. With the next attempt to deliver the shoulders, the unthinkable happened …decapitation. The doctor and I began to work through tears in our eyes. We just started to quietly cry. I thought I was seeing a horror film and that this could not be real. After the delivery was finished the obstetrician and I went into the locker room, put our arms around each other and cried. We did that repeatedly over the next few years!” (3)	

Maternity care providers reported that being outside the situation as a non-participant witness or listening to the affected women afterwards could also be traumatic (3, 4, 10, 11, 16, 28, 29, 33-36, 40, 45, 47, 51).

#### Disrespectful interactions in perinatal care

Poor communication and disrespectful interactions with women were reported as having a traumatic effect on care providers (13, 16, 28, 29, 51). Midwives reported high levels of stress in circumstances where they witnessed paternalistic interactions by healthcare professionals (51). Nurses and midwives described the impact of abusive care, such as violation of the woman’s bodily integrity and unnecessary roughness, witnessing or participating in procedures that were not in the woman’s or the baby’s best interest, and general interpersonal disrespect where the woman’s dignity was being ignored or her wishes overridden (3, 16, 28, 29, 51).

**Table:** 

“Care providers who ‘did not listen to women’ ‘provided little choice’, ‘pressured women to comply’, ‘did not gain consent’ and ‘used inappropriate language’.” (51)	

In addition, exposure to aggression or violence by the woman or by a family member was also reported as stressful and potentially traumatic (2, 13, 52, 53, 55, 57).

#### Conflicts between professional philosophies

Working within apposition of the ‘medical model’ of care and a ‘midwifery model of care’ was described as traumatic by some midwives (9, 40). Some felt that their care (management) was being questioned by other practitioners (16). This led to a feeling of being stuck in a conflict between different philosophies of care.

**Table:** 

“I find sometimes I get a bit, just lost, because … I know what I feel in terms of the natural side of things, but I’m working in a system where it’s not natural, and so you kind of get stuck between the two philosophies.” (40)	

#### Work environment

Several studies found that the working environment could intensify trauma for maternity care providers (9, 15, 25, 51, 52, 57). Stress and anxiety escalated in severe situations where care providers felt generally unsupported and had limited or delayed access to resources, when they needed to provide appropriate care (9, 46-48, 51). This included situations where staff shortages were an issue, staff were not experienced enough, or when it took a long time for theatre staff or an ambulance to arrive (4, 9, 13, 15, 46-48).

**Table:** 

‘‘We were all there scrubbed in theatre and basically it was – we were there for twenty-nine minutes (waiting for theatre staff to arrive). And it was just horrendous, you know. And we knew this baby had died, and we just were helpless you know, we couldn’t do anything about it at all.’’ (46)	

Obstetricians and midwives also reported other environmental factors that intensified stress around a traumatic birth, such as: staff conflict and bullying, culture of blame, personal criticism in case review meetings, incident report made about their management, censure by other professionals, performance review made by the employer after an adverse event, patient and disciplinary board complaints, discontented women, media coverage critical of care provided, and fear of litigation (2, 9, 15, 16, 25, 48, 51, 52, 57).

Midwives also described being disrespected or overruled as a professional when they had to watch and comply with care that was not evidence-based (9, 51).

### Impact of traumatic events on care providers

The impact of being involved in a traumatic event on care providers is summarised into two subcategories: *distressing emotions and physical symptoms*, and *impact on both professional and personal well-being*.

#### Distressing emotions and physical symptoms

Midwives, nurses, and obstetricians reported responses like stress, shock, sadness, anxiety, anger, horror, fear, guilt, disappointment, personal grief, and numbness that exemplified the experiences of attending traumatic births. Other emotions included self-blame, self-doubt, withdrawal from colleagues, and depression (3, 4, 7, 11, 12, 14, 18, 21-23, 25, 28, 29, 37, 39, 40, 43, 46, 48, 53, 54). Additionally, care providers experienced physical symptoms of headaches, fatigue, being irritable, sleep disorders, and generalised physical exhaustion while being involved in a traumatic birth (11, 14, 18, 42).

**Table:** 

“For weeks I could not get pictures of that dead baby girl out of my mind and had difficulty sleeping due to the nightmares.” (4)	

The intensity of these experiences often extended beyond their immediate impact, with long-lasting effects that were difficult for the participants to overcome (18). Guilt was a prominent emotion in many studies ( 3, 4, 7, 11, 12, 14, 21, 25, 28, 29, 39, 43, 46, 48, 53, 54). Guilt was perceived as a long-lasting emotion, remaining unresolved  (12, 43, 46). Care providers described it as feeling they had failed the women in their care and blamed themselves for not speaking up, for not being a better, more insightful, and knowledgeable professional  (3, 14, 43, 46, 48, 54). One study connected the emotions to not knowing how to face and manage traumatic events (39).

**Table:** 

“I put added stress on myself by beating myself up about the fact that could I have done something about it? That was the overwhelming feeling of what could I have done differently.’’ (46)	

However, nurses mentioned that when care could be offered in a respectful and considerate way, a potentially traumatic event like perinatal loss could also be a rewarding care experience that inspired change and growth in care providers (22).

#### Impact on both personal and professional well-being

Many care providers indicated or described that a traumatic event had a substantial adverse impact on their personal and professional well-being, even after a longer period of time (16, 18, 41, 46, 47, 50, 54). Most studies made no distinction between the effect on personal and professional well-being. Authors of one study suggested a larger impact of work-related events on day-to-day practice than effects on personal life (16). Another study showed an association between participants’ perceptions of trauma severity and the effect on their professional practice (41).

**Table:** 

“But it has affected [me]. Subconsciously it has affected, even though I try to resist. I will never be quite the same. And I do not feel that exuberant joy of going to work. And I am very, very afraid from time to time.” (54)	

Post-Traumatic Stress Disorder and symptoms of post-traumatic stress among care providers who cared for women with a traumatic childbirth was commonly reported, ranging from 3.2% to 75% of the participants being affected (2, 3, 4, 8, 10, 13-16, 24, 26-28, 30, 35, 36, 45, 49, 50, 53, 56, 57). This wide range mainly depended on selection bias of the population and cut-off scores for measuring post-traumatic stress.

A number of factors were associated with post-traumatic stress. Witnessing abusive care was associated with more severe post-traumatic stress than other types of trauma events (28, 45, 49). Care providers were also more likely to experience traumatic stress if they had an existing relationship with the woman (40, 45, 46), or had a personal traumatic birth experience (29, 45). Other associated factors were a strong reaction towards the event, such as feelings of guilt and horror, negative reactions from parents, the experience of insufficient support from local managerial staff, colleagues, friends and partner, as well as distressing experiences during debriefing (28, 29, 53). Furthermore, some studies showed that cultural factors like marginalised groups (black or minority) or certain geographical locations (Swiss midwives versus Japanese) were associated with a higher risk of post-traumatic stress (38, 50). This higher risk was also found for non-physicians rather than physicians (30) and NICU nurses more than midwives (15).

Positive associations were found between post-traumatic stress and factors such as: seniority, the number of traumatising events, quality of work life, burnout symptoms, compassion fatigue, negative cognition about the world, and negative self-cognition (10, 13, 34, 45).

In one study, midwives with high levels of distress reported impacts on their personal lives, such as becoming fearful about adverse events occurring to other people in their life or being vigilant for the safety of those around them (46). Other studies reported increased use of alcohol, drugs, nicotine or medication among nurses and obstetricians (2, 20, 30). For participating midwives, higher resilience and trait emotional intelligence scores were associated with reduced posttraumatic stress (36). Lower resilience significantly predicted posttraumatic stress (36).

A risk of burnout and more specifically of compassion fatigue was also associated with being involved in a traumatic childbirth event (1, 13, 20, 24, 42). Symptoms of compassion fatigue after a traumatic childbirth event adversely affected care providers’ professional quality of life (13). In one study, midwives reported higher scores of burnout than obstetricians, especially immediately following a traumatic childbirth event (39). However, sub-group analyses showed that this difference might be gender related with female care providers more at risk for burnout. In a qualitative study, midwives reported that they experienced high emotional exhaustion, due to the fact that they had an intensive workload and they were not allowed to take time off work (11). This was also found in quantitative studies where obstetricians and midwives reported emotional exhaustion (45, 49, 50). Years of work experience gave contradictory results for burnout, with both negative (1) and positive associations (13).

Clinical anxiety and depressive symptoms were reported to be related to traumatic events (8, 14, 26, 30). Midwives working in primary care seemed to be more at risk than hospital-based midwives (26). For both midwives and obstetricians, age and depression were positively associated (8, 14).

As a reaction to a traumatic event, some midwives, nurses and obstetricians reported that they had become a better clinician as a result of their experience (5, 6, 43, 57), this is described as posttraumatic growth (6, 57). Furthermore, midwives reported more resilience [6] and nurses reported a greater appreciation of life (5), while greater compassion towards others was reported among both (5, 6).

**Table:** 

“The traumatic births, such as shoulder dystocias, have helped me to realize how resilient I am. That I am brave. That I will not back down when things get hard or I feel threatened.” (6)	

### Changes in care providers’ practice

One of the effects of being involved in traumatic childbirth is that it can lead to changes in the practices of some care providers. Fear of litigation, disciplinary actions or public exposure seemed to contribute to these changes (3, 14, 51, 52, 54, 57). We summarised the changes that were reported in the studies in three subcategories: *Changes in the way care was provided*, *Changes in the interactions with women*, and *Changes to professional practice*.

#### Changes in the way care was provided

Care providers reported influences on the way they provided care to women because they had been involved in or witnessed a traumatic event (2, 3, 7, 9, 11, 26, 39, 41, 46, 54). Both midwives and obstetricians indicated practicing care in a more defensive manner by intervening more quickly, anticipating worst-case scenarios, performing more routine procedures or midwives consulting a physician more frequently (3, 7, 9, 11, 16, 31, 46). They reported being less able to work safely and effectively because of what had happened (44), and avoided certain situations, such as doing breech births, in order to control professional stress ([2, 39). Midwives who had experienced traumatic births, contemplated how they were more likely to have a shattered belief in the natural birth process (4, 31).

An opportunity to learn from the experience and improve future practice was also reported (5, 7, 16, 46). This included mentoring new colleagues and practical changes to procedures or protocols as well as personal changes, such as becoming more assertive (5, 7, 46).

**Table:** 

“It did affect my midwifery practice as after the event a woman had a panic attack. My heart just dropped, I stood back and called the emergency number. I knew she didn’t need the emergency team but I just lost confidence in myself and wanted some support.” (9)	

#### Changes in the interactions with women

Studies among midwives and obstetricians showed that the stress from experiencing traumatic events could result in withdrawing emotionally in their contact with the parents or distancing from engagement with women (14, 39, 44, 50). Studies reported that it could be challenging and difficult to communicate with families who have experienced a traumatic event (20, 40) and to continue to care for them (23). Positive changes for midwives included being more assertive as a woman’s advocate (5) and making more time for women (16).

**Table:** 

“At the end of the day, it allowed me to grow… I wouldn’t be where I am right now if it [event] wouldn’t have happened… maybe it even made me a better person… a better midwife”. (16)	

#### Changes to professional life

Many studies found that maternity care providers made changes in their professional life because of the traumatic events they had experienced. Midwives, nurses, and obstetricians described how they changed to another area of maternity care, such as not working in the labour ward or doing night shifts, or working in out-patient care (9, 26, 35, 36, 45, 46, 49, 53, 54). Many midwives, nurses, and obstetricians reported that their traumatic childbirth experience led them to contemplate leaving maternity care or making definite changes in their careers, such as going back to nursing, into academia or taking an administrative position (2, 3, 4, 9, 16, 19, 29, 30, 35, 36, 42-46, 50, 54, 57). Care providers also reported taking sick leave following an exposure to a traumatic perinatal event (35, 36, 45, 49, 50, 53).

**Table:** 

“After that case this senior registrar, you know year after she finished, she never did obstetrics, because everything came back to her … She just did gynecology.” (21)	

### Support for care providers

In the studies where maternity care providers reported on support after a traumatic event, they described *formal support* organised by their health institution and *informal support* offered by family/friends/colleagues, with some participants reporting receiving no support, neither formal nor informal.

Many studies did not include information on measures of support for health personnel after experiencing a traumatic event at the labour ward (1, 8, 10, 13, 14, 17, 19, 20, 23, 24, 27-29, 33, 34, 36, 38, 39, 42, 45, 49, 51, 52).

#### Formal support

Care providers indicated that specific support in relation to trauma was needed (9, 12, 15, 25, 32, 50). With regards to formal support for staff, studies described different appreciation of the support on a continuum from fairly adequate to being totally insufficient (2-5, 9, 11, 12, 19, 21, 25, 30, 40, 46-48, 51, 55, 57). Some even reported that their environment was toxic or unsafe [4]. They indicated that traumatic events were ignored in their organisation and used terms like *abandoned* and *betrayed *(4, 11, 21, 40). Their emotional needs were not considered by management, and clinical debriefing or other support was difficult to access (9, 18, 25, 46-48).

**Table:** 

“There was no recognition that it might be difficult … there was no training... there was no debriefing … and I think that’s bad. You did it yourself … nobody cared if you got so psychiatrically disturbed you threw yourself off the roof the following week.” (37)	

Debriefing was described in several studies as helpful (3, 4, 9, 12, 21) and, indeed, care providers acknowledged the importance of psychological debriefing from a mental health worker (8). In others however, this was not appreciated (55, 57). Professional supervision from someone with knowledge of childbirth, preferably from outside of the organisation, was viewed as paramount to lessening the trauma (9, 25, 55). In two studies, midwives and nurses mentioned that it was beneficial if a wider team of obstetricians, other nurses and midwives, anaesthesia staff, and neonatologists came together to debrief after a traumatic birth (3, 4). The presence of a support protocol or strategy in the hospital was rare (2, 26), but when available, it was reported as helpful (2, 26).

Participants in several studies suggested the development of educational and supportive interventions to prepare maternity care providers to cope with the emotional content of their work in the face of trauma (2, 12, 14, 15, 18, 22, 24, 35, 39, 47, 50). These interventions could also be part of the education programs for midwives, nurses and obstetricians (2). They could include building resilience, communication skills, and the ability to respond in the face of adversity (15). Additionally, being allowed to express emotions in the work context was valued and mentioned as helpful (2, 3) as well as allowing time to recover after a traumatic event (11, 15). Another study suggested that it could also be helpful to visit the woman afterwards to discuss the birth (3).

#### Informal support

Support by colleagues, partners and family was most common (2, 11, 16, 18, 21, 22, 26, 30-32, 35, 37, 44, 46, 51, 54). Most care providers preferred peer-support from direct colleagues as they could help to reconstruct and understand the event (2, 12, 18, 22, 26, 31, 37, 46, 50, 54). Support from colleagues who had experienced traumatic events themselves was mentioned as being especially valuable (50). In one study, when compared to obstetricians, midwives received more support from colleagues (44). Not getting support from colleagues was mentioned as worsening the traumatic event. Being blamed or mocked by colleagues had a devastating impact, adding to feelings of guilt and isolation (18, 31, 43, 48).

**Table:** 

“She felt completely unsupported... and then attacked for what she had done. Not by the consultant who she spoke to, but how the culture was. It was really difficult.” (54)	
“I felt love from my colleagues. Got loving support, phone calls home, checking again and again.” (54)	

## Discussion

In this scoping review, we mapped research investigating the impact of adverse events during the perinatal period on maternity care providers and how these experiences affected their well-being and professional practice. The 17 countries represented in this review were predominately high-income countries, except for one middle-income and one low-income country. Among the participants, midwives were the largest number of maternity care providers. This reflects maternity care service provision in high-income countries. Most studies were descriptive, with a mix of qualitative and quantitative designs. The majority of the included studies explored the effect of traumatic events during childbirth, whereas events during pregnancy or the postpartum period were scarcely reported.

The results of our study indicate that adverse events during childbirth have a serious impact on care providers. Wu [32] was one of the first who used the term “second victim” for care providers who were seriously affected by making an error or causing injury/death to an individual for whom they cared (the first victim). Since then, it has been used in a broader sense also including the possible traumatic impact on care providers caused by observing an adverse events or hearing about it [8]. Our exploration of the impact of adverse perinatal events on maternity care providers showed that trauma can originate from caring for or observing a woman with childbirth complications, which may or may not have had devastating outcome(s), such as maternal or infant death.

In addition, a few studies showed that missing a diagnosis or doubting a medical decision may also have a severe impact. Witnessing disrespectful interactions from colleagues to women was also reported as having a traumatic effect on care providers. Leinweber et al. [33] described this as care-related interpersonal birth trauma. In particular, midwives and nurses reported being highly stressed when they witnessed women being exposed to undignified care, such as not being listened to, a lack of shared decision making, a roughness during procedures or non-evidenced based care. The World Health Organization (WHO) acknowledges that disrespectful and undignified care is unacceptable, yet prevalent in many health care facilities across the globe, which keeps women from accessing individualised respectful maternity care services [34]. When women experience disrespectful care or a previous traumatic birth they may choose to avoid healthcare services and birth on their own, thereby plan to birth without the care of a maternity care provider [35]. This can be unsafe for both the women and her baby. Providing respectful maternity care to all women needs to be a priority for health service providers as it has wider implications for sustainability [36]. When women receive appropriate care during pregnancy, childbirth, and the postpartum period, they are less likely to experience complications that could lead to long-term health problems or even death. This reduces the burden on healthcare systems and contributes to the overall well-being of society, which is an important aspect of social sustainability. It promotes gender equality by ensuring that women have access to quality care and supports the well-being of future generations by ensuring that children are born healthy and have a strong foundation for growth and development [37]. Furthermore, the emotional toll of these events on maternity care professionals can add to the challenges related to their working conditions, such as long hours, understaffing, insufficient support from management and lack of resources [38]. These factors can result in job dissatisfaction, feelings of frustration and disillusionment, which can lead to some maternity care professionals seeking alternative roles within healthcare or may choose to leave their professions [10, 11, 14, 19, 23–25, 39]. The latter is of particular concern for midwives who are acknowledged as playing a critical role in maternity care practice provision during a global midwifery shortage crisis as outlined by the United Nations Population Fund [40].

The diverse origin of traumatic impact of severe birth events suggests that it is not only the nature of the event itself that is traumatising for care providers, but also the personal perception of the care provider or the way women are treated during care. Severe events seem to be less traumatising for women and care providers, when respect and good interaction between care providers and a woman can be established [41].

This implies that an important approach in preventing the traumatic impact of events on both care providers and women is respectful and dignified care. Organisation like the WHO, the International Confederation of Midwives (ICM) and International Federation of Gynecology and Obstetrics (FIGO) offer guidance and toolkits to establish respectful care [41–43].

In addition, participants in several studies suggested the development of educational programs to prepare maternity care providers for coping with the emotional content of their work. They advised that these programs could also be integrated in the curricula of future maternity care providers. Various programs have been developed and successfully tested [39, 44–46]. However, most of the programs seem to focus on only one professional group, such as midwives or obstetricians. An interprofessional approach may enhance understanding of the diversity in reactions and the impact of one’s own behaviour on colleagues with other backgrounds. Other measures to prevent traumatic impact of severe events that were reported in our study are direct and supporting responses of colleagues and management. Although informal support seems more available and is much appreciated, formal support is of additional value in recognising the reality of the impact and its consequences. A recent evaluation study on a formalised peer support program in two Danish hospital departments showed positive results [46]. The participating midwives, physicians, and nursing assistants agreed that it provided insight into how other people may react to adverse events was beneficial for themselves. The program encouraged an open and compassionate culture, attentiveness to the wellbeing of colleagues, and created a safe space for sharing. Still, the buddy system requires continuous maintenance and visibility. The program consists of a 2-h seminar about second victims and self-selected buddies to provide peer support after adverse events.

One of the included studies indicated that there might be a difference in impact of severe events between obstetricians and midwives. Sub-analyses suggested that gender might play a role with persons identifying as female at a higher risk of traumatic impact. This is a relevant aspect, in a domain where maternity healthcare is dominated by female care providers. However, this needs further exploration, as it might be related to other factors more prevalent in a female population, such as personal traumatic experience when giving birth themselves [8, 24, 47].

Future research should explore which maternity care providers are mostly at risk for the impact of traumatic events and under which conditions. Additionally, there is a need for research that examines theoretically-sound interdisciplinary interventions that can contribute to the prevention of perinatal traumatic events for women and maternity care providers.

### Strengths and weaknesses

A strength is that we rigorously followed the six-stage framework for scoping reviews developed by Arksey and O’Malley [26], refined by Levac et al. and the Joanna Briggs Institute [27, 28]. We also performed a wide search to identify papers relevant for the topic of our scoping review, including many databases, following up on specific authors, and consulting experts. Nevertheless, we may not have identified all relevant articles, as traumatic events in the perinatal period come with many different terminologies and include a broad range of events, while trauma is also used for many other aspects of childbirth, such a perineal lesions.

## Conclusion

The results from our scoping review show that the impact of traumatic perinatal events on maternity care providers is severe and far reaching. Although some care providers suggest that they became better clinicians as a result of what happened, many described the distressing effects on their psychological and physical health making them move position or resign from their employment in maternity care. Not getting adequate support from their organisation was something that came up frequently and strengthened the negative impact of the event.

With the current shortages in maternity care staff and the importance of a sustainable maternity workforce for the future, the impact of traumatic perinatal events requires serious consideration of how to maintain care providers wellbeing and positive engagement when conducting their profession.

### Supplementary Information

**Additional file 1: Supplement 1.** Search terms for this scoping review.

**Additional file 2.** Reference list of included papers.

**Additional file 3.** Characteristics of included studies.

**Additional file 4.** Questions for the content analysis.

## Data Availability

All data generated or analysed during this study are included in this published article and its supplementary information files.
