# Supplementary material for: The impact of severe perinatal events on maternity care providers: a scoping review
Source: BMC Health Serv Res. 2024 Feb 7;24:171. doi: 10.1186/s12913-024-10595-y (PMC10848539; doi:10.1186/s12913-024-10595-y)
Supplement: Supplementary file 1 — Additional file 1: Supplement 1. Search terms for this scoping review. [file 12913_2024_10595_MOESM1_ESM.docx]

Supplement 1. Search terms for this scoping review.

| **Population** |  | **Concept** |  | **Context** |
| --- | --- | --- | --- | --- |
| Maternity care provider | AND | Impact of severe event | AND | Maternity care |
| Maternity staff  Midwifery [MeSH]  Obstetrics [MeSH]  Obstetric nursing [MeSH]  Nurse Midwives [MeSH] |  | “Birth environment”  “Attitude of health personnel” [MeSH]  “Childbirth experiences”  Emotions [MeSH]  “Personal satisfaction” [MeSH]  “Burnout, professional”  “Perception”  “Decision Making” [MeSH]  “Delivery of health care” [MeSH]  “Stress, psychological” [MeSH]  “Psychological trauma” [MeSH]  “Pregnancy outcomes” [MeSH]  Trauma  Tokophobia  “Fear of (childbirth, birth)” |  | “Hospitals, maternity” [MeSH]  “Birthing centers” [MeSH]  “Birth setting"[MeSH]  "Home Childbirth"[MeSH]  “Maternal Health Services” [MeSH]  “Perinatal care” [MeSH]  “Maternity hospitals”  “Maternity led-units”  “Models of birth”  “Parturition” [MeSH]  “Postpartum period” [MeSH]  “Obstetric labor complications” [MeSH]  “Pregnancy complications” [MeSH] |
