# Supplementary material for: The impact of severe perinatal events on maternity care providers: a scoping review
Source: BMC Health Serv Res. 2024 Feb 7;24:171. doi: 10.1186/s12913-024-10595-y (PMC10848539; doi:10.1186/s12913-024-10595-y)
Supplement: Supplementary file 2 — Additional file 2. Reference list of included papers. [file 12913_2024_10595_MOESM2_ESM.docx]

**Reference list of included papers**

| **#** | **Reference** | **Source*** |
| --- | --- | --- |
|  | Amir Z, Reid AJ. Impact of traumatic perinatal events on burnout rates among midwives. Occup Med 2020;70(8):602-605. doi: 10.1093/occmed/kqaa156. | DS |
|  | Baas MAM, Scheepstra KWF, Stramrood CAI, Evers R, Dijksman LM, van Pampus MG. Work-related adverse events leaving their mark: a cross-sectional study among Dutch gynecologists. BMC Psychiatry 2018;18(1):73. doi: 10.1186/s12888-018-1659-1. | E |
|  | Beck CT, Gable RK. A mixed methods study of secondary traumatic stress in labor and delivery nurses. J Obstet Gynecol Neonatal Nursing 2012;41(6):747-60. doi: 10.1111/j.1552-6909.2012.01386.x. | SR |
|  | Beck CT, LoGiudice J, Gable RK. A mixed-methods study of secondary traumatic stress in certified nurse-midwives: shaken belief in the birth process. J Midwifery Womens Health 2015;60(1):16-23. doi: 10.1111/jmwh.12221. | SR |
|  | Beck CT, Eaton CM, Gable RK. Vicarious Posttraumatic Growth in Labor and Delivery Nurses. J Obstet Gynecol Neonatal Nursing 2016;45(6):801-812. doi: 10.1016/j.jogn.2016.07.008. Epub 2016 Oct 5. PMID: 27718368. | RL |
|  | Beck CT, Rivera J, Gable RK. A mixed-methods study of vicarious posttraumatic growth in certified nurse-midwives. J Midwifery Womens Health 2017;62(1):80-87. doi: 10.1111/jmwh.12523. | SR |
|  | Becker J, Becker C, Abeysekera R, Moir J, Gray M, Shimwela M, Oprescu F. Silent tears of midwives: 'i want every mother who gives birth to have her baby alive' - a narrative inquiry of midwives experiences of very early neonatal death from Tanzania. Children 2023;10(4):1-15. doi:10.3390/children10040705. | DS |
|  | Ben-Ezra M, Palgi Y, Walker R, Many A, Hamam-Raz Y. The impact of perinatal death on obstetrics nurses: a longitudinal and cross-sectional examination. J Perinat Med 2014;42(1):75-81. doi: 10.1515/jpm-2013-0071. | SR |
|  | Calvert I, Benn C. Trauma and the effects on the midwife. International Journal of Childbirth 2015;5(2):100-112. doi:10.1891/2156-5287.5.2.100. | SR |
|  | Çankaya S, Dikmen HA. The relationship between posttraumatic stress symptoms of maternity professionals and quality of work life, cognitive status, and traumatic perinatal experiences. Arch Psychiatr Nursing 2020;34(4):251-260. doi: 10.1016/j.apnu.2020.04.002. | DS |
|  | Cankaya S, Erkal Aksoy Y, Dereli Yılmaz S. Midwives' experiences of witnessing traumatic hospital birth events: A qualitative study. J Eval Clin Practice 2021;27(4):847-857. doi: 10.1111/jep.13487. | DS |
|  | Cauldwell M, Chappell LC, Murtagh G, Bewley S. Learning about maternal death and grief in the profession: a pilot qualitative study. Acta Obstet Gynecol Scand 2015;94(12):1346-53. doi: 10.1111/aogs.12760. | DS |
|  | Cohen R, Leykin D, Golan-Hadari D, Lahad M. Exposure to traumatic events at work, posttraumatic symptoms and professional quality of life among midwives. Midwifery 2017;50:1-8. doi: 10.1016/j.midw.2017.03.009. | DS |
|  | Farrow VA, Goldenberg RL, Fretts R, Schulkin J. Psychological impact of stillbirths on obstetricians. J Matern Fetal Neonatal Medicine 2013;26(8):748-52. doi: 10.3109/14767058.2012.746953. | SR |
|  | Favrod C, Jan du Chêne L, Martin Soelch C, Garthus-Niegel S, Tolsa JF, Legault F, Briet V, Horsch A. Mental Health Symptoms and Work-Related Stressors in Hospital Midwives and NICU Nurses: A Mixed Methods Study. Front Psychiatry 2018;9:364. doi: 10.3389/fpsyt.2018.00364. | E |
|  | Fontein-Kuipers Y, Duivis H, Schamper V, Schmitz V, Stam A, Koster D. Reports of work-related traumatic events: A mixedmethods study. Eur J Midwifery 2018;2:18. doi: 10.18332/ejm/100611. | E |
|  | Goldbort J, Knepp A, Mueller C, Pyron M. Intrapartum nurses' lived experience in a traumatic birthing process. MCN Am J Matern Child Nursing 2011;36(6):373-80. doi: 10.1097/NMC.0b013e31822de535. | RL |
|  | Halperin O, Goldblatt H, Noble A, Raz I, Zvulunov I, Liebergall Wischnitzer M. Stressful childbirth situations: a qualitative study of midwives. J Midwifery Womens Health 2011;56(4):388-394. doi: 10.1111/j.1542-2011.2011.00030.x. | SR |
|  | Hildingsson I, Westlund K, Wiklund I. Burnout in Swedish midwives. Sex Reprod Healthcare 2013;4(3):87-91. doi: 10.1016/j.srhc.2013.07.001. | DS |
|  | Hutti MH, Polivka B, White S, Hill J, Clark P, Cooke C, Clemens S, Abell H. Experiences of Nurses Who Care for Women After Fetal Loss. J Obstet Gynecol Neonatal Nursing 2016;45(1):17-27. doi: 10.1016/j.jogn.2015.10.010. | SR |
|  | Javid N, Hyett JA, Homer CSE. The experience of vasa praevia for Australian midwives: A qualitative study. Women Birth 2019;32(2):185-192. doi: 10.1016/j.wombi.2018.06.020. | DS |
|  | Jonas-Simpson CF, Pilkington B, MacDonald C, McMahon E. Nurses’ experiences of grieving when there is a perinatal death. SAGE Open 2013:1 –11. doi: 10.1177/2158244013486116. | SR |
|  | Jones K, Smythe E. The impact on midwives of their first stillbirth*.* New Zealand College of Midwives Journal 2015;51:17–22. doi:10.12784/nzcomjnl51.2015.3.17-22. | RL |
|  | Katsantoni K, Zartaloudi A, Papageorgiou D, Drakopoulou M, Misouridou E. Prevalence of Compassion Fatigue, Burn-Out and Compassion Satisfaction Among Maternity and Gynecology Care Providers in Greece. Mater Sociomed 2019;31(3):172-176. doi: 10.5455/msm.2019.31.172-176. | DS |
|  | Kave YV, Sonti BSI, Morton DG, Sindiwe J. Supporting the needs of midwives caring for women with perinatal loss in South Africa. British Journal of Midwifery 2023;31(1): 16-22. doi: 10.12968/bjom.2023.31.1.16. | DS |
|  | Kerkman T, Dijksman LM, Baas MAM, Evers R, van Pampus MG, Stramrood CAI. Traumatic Experiences and the Midwifery Profession: A Cross-Sectional Study Among Dutch Midwives. J Midwifery Womens Health 2019;64(4):435-442. doi: 10.1111/jmwh.12946. | E |
|  | Komachi MH, Kamibeppu K, Nishi D, Matsuoka Y. Secondary traumatic stress and associated factors among Japanese nurses working in hospitals. Int J Nurs Pract 2012;18(2):155-63. doi: 10.1111/j.1440-172X.2012.02014.x. | RL |
|  | Leinweber J, Creedy DK, Rowe H, Gamble J. Responses to birth trauma and prevalence of posttraumatic stress among Australian midwives. Women Birth 2017a;30(1):40-45. doi: 10.1016/j.wombi.2016.06.006. | DS |
|  | Leinweber J, Creedy DK, Rowe H, Gamble J. A socioecological model of posttraumatic stress among Australian midwives. Midwifery 2017b;45:7-13. doi: 10.1016/j.midw.2016.12.001. | DS |
|  | Margulies SL, Benham J, Liebermann J, Amdur R, Gaba N, Keller J. Adverse Events in Obstetrics: Impacts on Providers and Staff of Maternity Care. Cureus 2020;12(1):e6732. doi: 10.7759/cureus.6732. | E |
|  | Minooee S, Cummins A, Foureur M, Travaglia J. Catastrophic thinking: Is it the legacy of traumatic births? Midwives' experiences of shoulder dystocia complicated births. Women Birth 2021a;34(1):e38-e46. doi: 10.1016/j.wombi.2020.08.008. | DS |
|  | Minooee S, Cummins A, Foureur M, Travaglia J. Shoulder dystocia: A panic station or an opportunity for post-traumatic growth? Midwifery 2021b;101:103044. doi: 10.1016/j.midw.2021.103044. | DS |
|  | Muliira RS, Bezuidenhout MC. Occupational exposure to maternal death: psychological outcomes and coping methods used by midwives working in rural areas. Midwifery 2015a;31(1):184-90. doi: 10.1016/j.midw.2014.08.005. | DS |
|  | Muliira RS, Sendikadiwa VB, Lwasampijja F. Predictors of death anxiety among midwives who have experienced maternal death situations at work. Matern Child Health J. 2015b;19(5):1024-32. doi: 10.1007/s10995-014-1601-1. | DS |
|  | Nicholls EM, Hermann RM, Giordano NA, Trotta RL. Secondary Traumatic Stress Among Labor and Delivery Nurses. MCN Am J Matern Child Nursing 2021;46(1):14-20. doi: 10.1097/NMC.0000000000000674. | E |
|  | Nightingale S, Spiby H, Sheen K, Slade P. Posttraumatic stress symptomatology following exposure to perceived traumatic perinatal events within the midwifery profession: The impact of trait emotional intelligence. J Adv Nursing 2018. doi: 10.1111/jan.13719. | DS |
|  | Nuzum D, Meaney S, O'Donoghue K. The impact of stillbirth on consultant obstetrician gynaecologists: a qualitative study. BJOG 2014;121(8):1020-8. doi: 10.1111/1471-0528.12695. | DS |
|  | Oe M, Ishida T, Favrod C, Martin-Soelch C, Horsch A. Burnout, Psychological Symptoms, and Secondary Traumatic Stress Among Midwives Working on Perinatal Wards: A Cross-Cultural Study Between Japan and Switzerland. Front Psychiatry 2018;9:387. doi: 10.3389/fpsyt.2018.00387. | E |
|  | Pastor Montero SM, Romero Sánchez JM, Hueso Montoro C, Lillo Crespo M, Vacas Jaén AG, Rodríguez Tirado MB. Experiences with perinatal loss from the health professionals' perspective. Rev Lat Am Enfermagem 2011;19(6):1405-12. doi: 10.1590/s0104-11692011000600018. | DS |
|  | Rice H, Warland J. Bearing witness: midwives experiences of witnessing traumatic birth. Midwifery 2013;29(9):1056-63. doi: 10.1016/j.midw.2012.12.003. | DS |
|  | Robinson KA, Johantgen ME, Storr CL, Gaitens JM, Atlas RO, Ogbolu Y. Cross-sectional study of the frequency and severity of traumatic childbirth events and how they affect maternity care clinicians. Journal of obstetric, gynecologic, and neonatal nursing 2023;52(1):84-94. doi: 10.1016/j.jogn.2022.08.006. | DS |
|  | Schrøder K, Larsen PV, Jørgensen JS, Hjelmborg JV, Lamont RF, Hvidt NC. Psychosocial health and well-being among obstetricians and midwives involved in traumatic childbirth. Midwifery 2016a;41:45-53. doi: 10.1016/j.midw.2016.07.013. | DS |
|  | Schrøder K, Jørgensen JS, Lamont RF, Hvidt NC. Blame and guilt - a mixed methods study of obstetricians' and midwives' experiences and existential considerations after involvement in traumatic childbirth. Acta Obstet Gynecol Scand 2016b;95(7):735-45. doi: 10.1111/aogs.12897. | DS |
|  | Schrøder K, Edrees HH, Christensen RD, Jørgensen JS, Lamont RF, Hvidt NC. Second victims in the labor ward: Are Danish midwives and obstetricians getting the support they need? Int J Qual Health Care 2019;31(8):583-589. doi: 10.1093/intqhc/mzy219. | SR |
|  | Sheen K, Spiby H, Slade P. Exposure to traumatic perinatal experiences and posttraumatic stress symptoms in midwives: prevalence and association with burnout. Int J Nurs Studies 2015;52(2):578-87. doi: 10.1016/j.ijnurstu.2014.11.006. | DS |
|  | Sheen K, Spiby H, Slade P. The experience and impact of traumatic perinatal event experiences in midwives: A qualitative investigation. Int J Nurs Studies 2016a;53:61-72. doi: 10.1016/j.ijnurstu.2015.10.003. | DS |
|  | Sheen K, Spiby H, Slade P. What are the characteristics of perinatal events perceived to be traumatic by midwives? Midwifery 2016b;40:55-61. doi: 10.1016/j.midw.2016.06.007. | DS |
|  | Sheen K, Goodfellow L, Balling K, Rymer J, Weeks A, Spiby H, Slade P. Which events are experienced as traumatic by obstetricians and gynaecologists, and why? A qualitative analysis from a cross-sectional survey and in-depth interviews. BMJ Open 2022;12(11):e061505. doi: 10.1136/bmjopen-2022-061505. | DS |
|  | Slade P, Sheen K, Collinge S, Butters J, Spiby H. A programme for the prevention of post-traumatic stress disorder in midwifery (POPPY): indications of effectiveness from a feasibility study. Eur J Psychotraumatology 2018;9(1):1518069. doi: 10.1080/20008198.2018.1518069. | DS |
|  | Slade P, Balling K, Sheen K, Goodfellow L, Rymer J, Spiby H, Weeks A. Work-related post-traumatic stress symptoms in obstetricians and gynaecologists: findings from INDIGO, a mixed-methods study with a cross-sectional survey and in-depth interviews. BJOG 2020;127(5):600-608. doi: 10.1111/1471-0528.16076. | DS |
|  | Toohill J, Fenwick J, Sidebotham M, Gamble J, Creedy DK. Trauma and fear in Australian midwives. Women Birth 2019;32(1):64-71. doi: 10.1016/j.wombi.2018.04.003. | DS |
|  | Wahlberg Å, Andreen Sachs M, Bergh Johannesson K, Hallberg G, Jonsson M, Skoog Svanberg A, Högberg U. Self-reported exposure to severe events on the labour ward among Swedish midwives and obstetricians: A cross-sectional retrospective study. Int J Nurs Studies 2017a;65:8-16. doi: 10.1016/j.ijnurstu.2016.10.009. | DS |
|  | Wahlberg Å, Andreen Sachs M, Johannesson K, Hallberg G, Jonsson M, Skoog Svanberg A, Högberg U. Post-traumatic stress symptoms in Swedish obstetricians and midwives after severe obstetric events: a cross-sectional retrospective survey. BJOG 2017b;124(8):1264-1271. doi: 10.1111/1471-0528.14259. | DS |
|  | Wahlberg Å, Högberg U, Emmelin M. The erratic pathway to regaining a professional self-image after an obstetric work-related trauma: A grounded theory study. Int J Nurs Studies 2019;89:53-61. doi: 10.1016/j.ijnurstu.2018.07.016. | DS |
|  | Wahlberg Å, Högberg U, Emmelin M. Left alone with the emotional surge - A qualitative study of midwives' and obstetricians' experiences of severe events on the labour ward. Sex Reprod Healthcare 2020;23:100483. doi: 10.1016/j.srhc.2019.100483. | DS |
|  | Wallbank S, Robertson N. Predictors of staff distress in response to professionally experienced miscarriage, stillbirth and neonatal loss: a questionnaire survey. Int J Nurs Studies 2013;50(8):1090-7. doi: 10.1016/j.ijnurstu.2012.11.022. | SR |
|  | Walker AL, Gamble J, Creedy DK, Ellwood DA. Impact of traumatic birth on Australian obstetricians: A pilot feasibility study. Aust N Z J Obstet Gynaecology 2020;60(4):555-560. doi: 10.1111/ajo.13107. | DS |

* Source of paper:

DS = database search

E = expert

RL = reference list

SR = systematic review
