# Supplementary material for: The impact of severe perinatal events on maternity care providers: a scoping review
Source: BMC Health Serv Res. 2024 Feb 7;24:171. doi: 10.1186/s12913-024-10595-y (PMC10848539; doi:10.1186/s12913-024-10595-y)
Supplement: Supplementary file 3 — Additional file 3. Characteristics of included studies. [file 12913_2024_10595_MOESM3_ESM.docx]

**Characteristics of included studies**

| **#** | **First author (publication year) *title***  **Country** | **Study design**  **Data collection method** | **Study aim** | **Study participants** | **Severe event** | **Measurement outcomes** |
| --- | --- | --- | --- | --- | --- | --- |
|  | Amir Z (2020)  *Impact of traumatic perinatal events on burnout rates among midwives.*  Ireland | Cross-sectional survey | To establish the prevalence of burnout among midwives in Ireland and whether exposure to traumatic perinatal events in work contributes to this. | 137 midwives (response rate 55%) from a tertiary referral maternity hospital. | Events for which the respondents ‘did not feel adequately prepared’ or that they ‘found upsetting or overwhelming’. | Two visual analogue scales that reflected the impact of the event and the resulting distress as a single score with a range from 0 (no distress) to 20 (maximum distress).  Copenhagen Burnout Inventory (CBI) |
|  | Baas MAM (2018) *Work-related adverse events leaving their mark: a cross-sectional study among Dutch gynecologists.*  Netherlands | Cross-sectional survey | To study the prevalence of work-related traumatic events and PTSD among ObGyns. To explore the coping and professional support after work-related adverse events. | 683 ObGyns, members of the  Dutch Society of Obstetrics & Gynecology. | Unanticipated adverse patient event, a medical error and/or a patient related injury. | Trauma Screening Questionnaire (TSQ)  Considering leaving their medical profession. |
|  | Beck CT (2012)  *A mixed methods study of secondary traumatic stress in labor and delivery nurses.*  USA | Mixed methods - survey with closed and open-ended questions | To determine the prevalence and severity of secondary traumatic stress in labor and delivery (L&D) nurses and to explore nurses’ descriptions of their experiences attending traumatic births. | 464 L&D nurses | Self-determined by participants. | Secondary traumatic stress Scale (STS*-*scale)  Description of experiences of attending one or more traumatic births.  Description of thoughts, feelings, and perceptions.  Description of impact on nursing practice. |
|  | Beck CT (2015)  *A mixed-methods study of secondary traumatic stress in certified nurse-midwives: Shaken-belief in the birth process.*  USA | Mixed methods - survey with closed and open-ended questions | To determine the prevalence and severity of secondary traumatic stress and explore the experience of nurse-midwives who attend traumatic births. | 473 certified nurse-midwives completed quantitative questions; 246 completed qualitative element. | Self-determined by participants. | Secondary traumatic stress Scale (STS*-*scale)  Description of experiences of attending one or more traumatic births.  Description of thoughts, feelings, and perceptions. Description of impact on midwifery practice. |
|  | Beck CT (2016) *Vicarious posttraumatic growth in labor and delivery nurses*  USA | Mixed methods - survey with closed and open-ended questions | To investigate vicarious posttraumatic growth in labor and delivery nurses who cared for women during traumatic births. | 467 labor and delivery nurses who completed the quantitative portion with 295 (63%) who completed the qualitative portion. | Self-determined by participants. | Posttraumatic Growth  Inventory (PTGI)  Core Beliefs Inventory (CBI)  Description of experiences of any positive changes in beliefs or life as a result of caring for women during traumatic births. |
|  | Beck CT (2017)  *A mixed-methods study of vicarious posttraumatic growth in certified nurse-midwives*  USA | Mixed methods - survey with closed and open-ended questions | To investigate vicarious posttraumatic growth in certified nurse-midwives (CNMs) who have struggled after attending traumatic births. | 425 CNMs who completed the quantitative portion and 315 (74%) who completed the qualitative portion. | Self-determined by participants. | Posttraumatic Growth Inventory  Core Beliefs Inventory  Description of experiences of any positive changes in beliefs or life as a result of attending traumatic births. |
|  | Becker J (2023) *Silent tears of midwives: 'i want every mother who gives birth to have her baby alive'*  Tanzania | Qualitative study – individual interview | To explore how midwives are impacted by and cope with high rates of very early neonatal deaths. | 21 midwives | Very early neonatal death | Impact of and coping with high rates of very early neonatal deaths.  Insights and local solutions that may reduce very early neonatal deaths in limited resource settings.  How to create awareness and garner support for midwives and their work in low resource settings. |
|  | Ben-Ezra M (2014) *The impact of perinatal death on obstetrics nurses: a longitudinal and cross-sectional examination.*  Israel | Cross-sectional survey with longitudinal follow-up | To test the hypothesis that exposure to perinatal death leads to exacerbation in mental health and well-being. | 27 nurse-midwives exposed to perinatal death and 63 nurses as controls | Perinatal death | Impact of Event Scale – Revised (IES-R)  Center for Epidemiologic Studies Depression Scale (CES-D)  Psychosomatic Problems Scale (PSP)  Life satisfaction  Scale of Positive and Negative Experience (SPANE) |
|  | Calvert I (2015) *Trauma and the Effects on the Midwife.*  New Zealand | Qualitative study - individual interviews | To explore the effects of a traumatic practice experience on the midwifery practitioner. | 16 practicing midwives who self-identified as having experienced a traumatic practice experience. | Self-determined by participants. | Description of traumatic practice experience and how this event was perceived.  Meaning of the event for the  Individual. |
|  | Çankaya S (2020)  *The relationship between posttraumatic stress symptoms of maternity professionals and quality of work life, cognitive status and traumatic perinatal experiences.*  Turkey | Cross-sectional survey | To determine the relationship between posttraumatic stress symptoms of maternity nursing/midwives and their quality of work life, cognitive distortions and traumatic perinatal experiences. | 266 maternity nurses and midwives employed in maternity units of hospitals. | Such as attending or witnessing:  Very difficult birth (with a vacuum or forceps), death of mother or baby, baby with an abnormality, stillbirth, maltreatment of the attending physician (cursing, insult, etc.) against the woman, disregarding the requests of the woman. | Impact of Event Scale–Revised (IES-R)  Professional Quality of Life Scale (ProQOL R-IV)  Post-Traumatic Cognitions Inventory (PTCI)  Criterion A of the DSM-IV-TR for PTSD |
|  | Çankaya S (2021) *Midwives' experiences of witnessing traumatic hospital birth events: A qualitative study.*  Turkey | Qualitative study - individual interviews | To investigate in detail the traumatic birth experiences of midwives in the delivery rooms, and their attitudes, reactions, and coping strategies. | 29 midwives, who work in labour and birth room. | A birth event experienced that threatened the life of the mother and baby. | Emotions, actions, and influences related to the traumatic event, their coping strategies, and support systems were discussed. |
|  | Cauldwell M (2015) *Learning about maternal death and grief in the profession: a pilot qualitative study.*  UK | Qualitative study - individual interviews | To explore the impact of maternal death on maternity professionals, and their related professional and personal needs. | 14 participants (4 midwives; 5 doctors in training; 5 consultant obstetricians) working in one hospital. | Maternal death | Interviewees were encouraged to describe and reflect on preparation for, or their experiences of, maternal death in order to determine: (i) whether it was possible to be prepared by formal training or guidance and (ii) how staff should best be supported after a maternal death occurs. |
|  | Cohen R (2017)  *Exposure to traumatic events at work, posttraumatic symptoms, and professional quality of life among midwives.*  Israel | Cross sectional survey | To study midwives’ professional quality of life and traumatic experiences through the measurement of compassion fatigue and compassion satisfaction. | 93 midwives working at four medical centres. | Injury to oneself, injury to others, perceived life threat, perceived threat to the life of others, feelings of helplessness, terror, and exposure to horrifying images experienced at work. | PTSD symptom scale – self report (PSS-SR)  The Professional Quality of Life scale (ProQOL) composed of three sub-scales: compassion satisfaction (CS), burnout (BO) and compassion fatigue/secondary trauma symptoms (STS) |
|  | Farrow VA (2012) *Psychological impact of stillbirths on obstetricians.*  USA | Cross-sectional survey | To assess the psychological impact on US obstetricians when they care for women who have suffered a stillbirth and explore whether demographic and practice variables were related to the extent of psychological impact for obstetricians following stillbirth. | 499 obstetricians of whom 365 currently practiced obstetrics. | Stillbirth | Experience of a variety of emotional responses:   - Grief - Depression - Self-blame - Self-doubt - Feelings of guilt - Fear of legal action - Inability to sleep for two or more weeks - PTSD   Current performance regarding maternal/family counselling, post-delivery of a stillbirth. |
|  | Favrod C (2018) *Mental Health Symptoms and Work-Related Stressors in Hospital Midwives and NICU Nurses: A Mixed Methods Study.*  Switzerland | Mixed- methods - survey with one open-ended question | To compare mental health symptoms in hospital midwives and NICU nurses, and to identify and compare work-related traumatic and non-traumatic stressors for both professional groups. | 122 midwives and 91 NICU nurses working in two university hospitals. | No definition  Description of work-related stressors encountered at work. | Secondary Traumatic Stress Scale (STSS)  Hospital Anxiety and Depression Scale (HADS)  Maslach Burnout Inventory |
|  | Fontein Y (2018) *Reports of work-related traumatic events: A mixed- methods study.*  Netherlands and Belgium | Mixed- methods study -  survey and individual interviews | To study the effect and impact on midwives of being involved or witnessing traumatic work-related events. | 104 practicing midwives in the quantitative study.  12 practicing midwives in qualitative study. | Self-determined by participants. | i) Three items to describe the traumatic event and the influences on professional and personal life  ii) One item categorising responses to the event  iii) Two items to measure the influence on professional and personal life  iv) Five items measuring stress with a 5-point rating scale.  Three or more scores of ≥4 were considered clinically relevant for post-traumatic stress (PTSD). The five items stem from the DSM-IV PTSD categorisation. |
|  | Goldbort J (2011) *Intrapartum nurses’ lived experience in a traumatic birthing process.*  USA | Qualitative study - individual interviews | To describe nurses’ participation in an unexpected/ traumatic birthing process to ascertain what impact this experience had on the nurse. | 9 intrapartum nurses | (1) An instrumentally assisted vaginal birth either by forceps and/or by a vacuum extractor  that failed and resulted in an emergency caesarean birth;  (2) Any unexpected complications, such as shoulder  dystocia and postpartum haemorrhage;  (3) An infant who required unexpected neonatal resuscitation;  (4) Any birthing experience that was perceived by the nurse as unexpected/traumatic. | Description of experience of participating in an unexpected/traumatic birth.  How did that experience make you feel? |
|  | Halperin O (2011) *Stressful childbirth situations: A qualitative study of midwives*  Israel | Qualitative study - individual interviews | To explore clinical life-threatening childbirth situations, which midwives perceive as extremely stressful, and to identify how midwives cope with those experiences. | 18 midwives employed in 6 labor and delivery units in hospitals | Extremely stressful events in situation with mothers and/or newborns. | Five open-ended narrative questions were asked to allow the participants to tell their stories and describe their feelings, coping strategies, and support systems. |
|  | Hildingsson I (2013)  *Burnout in Swedish midwives*  Sweden | Cross-sectional survey | To investigate the level of burnout amongst midwives and their attitude towards leaving the profession. | 475 of a random sample of 978 midwives (48.6% response rate) who are members of Swedish Midwifery Association. | Experience of a critical situation such as intrauterine foetal death, and threatful patients or relatives. | Copenhagen Burnout Inventory (CBI)  Question on considering leaving work. |
|  | Hutti MH (2016) *Experiences of nurses who care for women after fetal loss*  USA | Qualitative study - focus groups | To examine the experiences of, meaning for, and personal consequences for obstetric, emergency, and surgical nurses caring for women after fetal death and to determine how these nurses use Swanson’s caring processes in providing such care. | 24 registered nurses working in the obstetric, surgery, or emergency departments. | Fetal death | Participants were asked to describe the meaning of caring for a patient/family that has had a foetal loss and the nature of the care provided. In addition, they were asked what was needed to enhance their ability to provide care to patients/families with a foetal loss. |
|  | Javid N (2019)  *The experience of vasa praevia for Australian midwives: A qualitative study.*  Australia | Qualitative study - individual interviews | To investigate the experience of midwives looking after women with undiagnosed vasa praevia.  Further experience of involvement in neonatal death or near miss due to vasa praevia. | 20 midwives who had looked after at least one woman with vasa praevia between 2010 and 2016. | Experience of vasa praevia | How was the experience for you?  What was the woman’s reaction?  How was the baby when born?  How was it like for the others who were involved in that case? |
|  | Jonas-Simpson CF (2013)  *Nurses’ experiences of grieving when there is a perinatal death.*  Canada | Qualitative study - individual interviews | To explore obstetrical and neonatal nurses’ experiences of grieving when caring for families who experience loss after perinatal death. | 5 obstetrical nurses and 1 neonatal intensive care nurse who cared for bereaved families. | Perinatal death | Concepts of meaning, patterns of relating, and transformation. |
|  | Jones K (2015)  *The impact on midwives of their first stillbirth.*  New Zealand | Qualitative study - individual interviews | To understand the midwife’s experiences in relation to the loss of a baby. | 5 self-employed midwives | Stillbirth | The thoughts and feelings associated with the event. |
|  | Katsantoni K (2019)  *Prevalence of compassion fatigue, burnout and compassion satisfaction among maternity and gynecology care providers in Greece.*  Greece | Cross-sectional study | To investigate the level of risk for compassion fatigue/secondary traumatic stress and burnout for maternity and gynaecology care providers and levels compassion satisfaction and possible effects of personal and work-related factors. | 80 registered nurses or midwives, and 41 nurse or midwives’ assistants working at public hospitals. | Not defined | Professional Quality of Life Scale (ProQOL R-IV)  Professional with three discrete scales: compassion satisfaction (CS), burnout (BO) and compassion fatigue/ secondary trauma (CF/STS)  Desire of the same career for their children  Choice of the same career again for themselves |
|  | Kave YV (2023)  *Supporting the needs of midwives*  *caring for women with perinatal*  *loss in South Africa*  South Africa | Qualitative study - individual interviews | To explore the coping behaviours and support needs of midwives caring for women with perinatal loss. | 13 midwives | Perinatal loss | Coping mechanisms in relation to perinatal loss.  Views on support from  Management.  Need for psychological and emotional support. |
|  | Kerkman T (2019) *Traumatic Experiences and the Midwifery Profession:*  *A Cross-Sectional Study Among Dutch Midwives*  Netherlands | Cross-sectional survey | To investigate the prevalence of work-related traumatic events, PTSD, anxiety, and depression; the differences between midwives working in primary care and midwives working in secondary or tertiary care (hospital setting) and the support midwives would like to receive after experiencing a work-related adverse event. | 691 midwives, members of the Dutch Organization of Midwives, working in the Netherlands. | What situations at work do you perceive being emotionally most stressful?   - bringing bad news - critically ill moments of a patient or baby - missing a diagnosis - death of the mother or child - doubt regarding the decision that has been made | Trauma Screening Questionnaire (TSQ)  Hospital Anxiety and Depression Scale (HADS)  Four questions regarding emotionally exhausting moments at work, and how support could best be developed. |
|  | Komachi MH (2012) *Secondary traumatic stress and associated factors among Japanese nurses working in hospitals*  Japan | Cross-sectional survey | To evaluate the prevalence and factors associated with secondary traumatic stress among general hospital nurses. | 176 nurses (52.1% response rate), with 159 (90.3%) participants reported encountering a traumatic event while providing nursing care. | Cared for a pregnant woman or a woman in childbirth who was in serious condition  Cared for a pregnant woman or a woman in childbirth who died  Cared for a newborn or infant in serious condition  Cared for a pregnant woman who had an abortion or  miscarriage after 6 months of pregnancy | Eysenck Personality Questionnaire-Revised (EPQ-R)  Accepting Responsibility subscale of the Stress Coping Inventory (SCI)  Social Support Questionnaire (SSQ)  Impact of Event Scale-Revised (IES-R) |
|  | Leinweber J (2017a) *Responses to birth trauma and prevalence of posttraumatic stress among Australian midwives.*  Australia | Cross-sectional survey | To assess different types of birth trauma, peritraumatic reactions and prevalence of posttraumatic stress symptoms | 687 midwives, members of Australian College of Midwives | Traumatic Events in Perinatal Care List (TEPCL) assessing different types of traumatic events witnessed by care providers during labour and birth.  Research that described nurses and midwives’ experiences of witnessing traumatic birth events and research into traumatic childbirth experiences with women were used to create a list of care related interpersonal and non-interpersonal trauma event features. | Midwives were asked to indicate whether or not (yes/no) they recalled feeling fear, horror, and helplessness during or shortly after the traumatic event. In addition, they were asked to indicate whether or not (yes/no) they recalled feeling anger or guilt during or shortly after the index birth trauma event.  PTSD Symptom Scale Self-Report version (PSS-SR) |
|  | Leinweber J, et al. (2017b)  *A socioecological model of posttraumatic stress among Australian midwives.*  Australia | Cross-sectional survey | To develop a model of personal, trauma and work-related risk factors for post-traumatic stress disorder for Australian midwives who witness traumatic birth | 601 midwives, members of Australian College of Midwives | The notion of trauma is "in the eye of the beholder”, which means that if you recall an experience/event around a labour and birth as traumatic, then it was traumatic.’  Using the Traumatic Events In Perinatal Care List (TEPCL) five categories of witnessed traumatic birth event features were listed (1) death or (2) severe injury of mother or baby (3) abusive care (4) involvement in suboptimal care and (5) disrespect of women's dignity. | Seven categories of peritraumatic distress (1) fear (2) horror (3) helplessness (4) anger (5) guilt (6) responsibility (7) powerlessness were asked to endorse (yes/no).  PTSD Symptom Scale Self-Report version (PSS-SR)  ‘Empathic concern’ and ‘personal distress’ subscales of the Interpersonal Reactivity Index (IRI) |
|  | Marguiles SL (2020) *Adverse events in obstetrics: Impacts on providers and staff of maternity care.*  USA | Cross-sectional survey | To determine the frequency of maternity health employee experiences with maternal and perinatal/ neonatal adverse outcomes and gain a deeper understanding of how these experiences impact the providers. | 105: physicians (OB/GYN and anaesthesia) 43, midwives 6, nurses 35, and hospital employees in foodservice and housekeeping 21 | Experiences with maternal and perinatal/neonatal adverse outcomes. | Patient Health Questionnaire-4  Primary Care Post-Traumatic Stress Disorder Screen (PC-PTSD) |
|  | Minooee S (2021a) *Catastrophic thinking: Is it the legacy of traumatic births? Midwives’*  *experiences of shoulder dystocia complicated births.*  Australia | Qualitative study - individual interviews | To explore the impact of SD, as a birth trauma, on midwives’ orientation towards normal births and on their clinical practice and the factors which may deteriorate or improve the experience of SD. | 25 midwives experiencing at least one case of shoulder dystocia were invited to the study through the Australian College of Midwives (ACM). | A birth complicated by shoulder dystocia | Feelings during and after the birth; emotions and thoughts developed after the birth; potential impact of these thoughts on the next births. |
|  | Minooee S (2021b) *Shoulder dystocia : a panic station or an opportunity for growth?*  Australia | Qualitative study -individual interviews | To explore the impact of experiencing shoulder dystocia on clinical practice of midwives. | 25 midwives experiencing at least one case of shoulder dystocia were invited to the study through the Australian College of Midwives (ACM). | A birth complicated by shoulder dystocia | Using video webcam to observe the interviewee’s visual cues (facial expressions or body movements), and to reflect on the feelings of the interviewee.  What were you feeling when the baby was born?  Can you tell me about your feelings in the next births?  Can you tell me about the support you received after that birth? |
|  | Muliira RS (2014) *Occupational exposure to maternal death: Psychological outcomes and coping methods used by midwives working in rural areas.*  Uganda | Cross-sectional survey | To determine death anxiety predictors among midwives who have experienced maternal death to enable recommendations on interventions for coping with distress | 238 midwives working in two rural districts of Uganda. | Occupational exposure to maternal death. | Death Distress Scale (DDS)  Brief COPE Scale |
|  | Muliira RS (2015)  *Predictors of death anxiety among midwives who have experienced maternal death situations at work.*  Uganda | Cross-sectional survey | To determine the predictors of death anxiety among midwives who have experienced maternal death at work in order to recommend interventions to facilitate effective coping with the distress. | 224 midwives working in two rural districts of Uganda. | Occupational exposure to maternal death. | Death Distress Scale (DDS)  Brief COPE Scale  Perceived Wellbeing Scale (PWS) |
|  | Nicholls EM (2021) *Secondary traumatic stress among labor and delivery nurses.*  USA | Cross-sectional survey | To describe the prevalence and severity of secondary traumatic stress (STS) among labour and delivery nurses. | 144 labour and delivery nurses (response 50%) | Have you ever witnessed a traumatic birth in your position as a labour and delivery nurse? Approximately, how many times have you experienced a traumatic birth in your position as a labour and delivery nurse? | Secondary Traumatic Stress Scale (STSS)  Have you considered leaving your position in labour and delivery because of a traumatic birth experience?  Have you ever called out sick because of stress related to a traumatic birth experience in your position as a labour and delivery nurse?  Have you ever asked for a change in assignment in order to avoid a potential trauma after experiencing a traumatic birth?  Participants were also asked what resources or sources of support they utilized in relation to a traumatic birth experience. |
|  | Nightingale (2018)  *Posttraumatic symptomatology following exposure to perceived traumatic perinatal events within the midwifery profession: The impact of trait emotional intelligence.*  UK | Cross-sectional survey | To explore factors associated with, and predictors of posttraumatic stress in midwives with the potential moderating effects of trait emotional intelligence | 113 midwives from NHS hospitals Trusts having been involved in a perceived traumatic perinatal event within the last 10 years. | A perinatal event that included actual or threatened death or serious injury to the mother and/or child. | PTSS with the Impact of Event Scale-Revised (IES-R) |
|  | Nuzum D (2014)*The impact of stillbirth on consultant obstetrician gynaecologists: a qualitative study.* Ireland | Qualitative study – individual interviews | To explore the personal and professional impact of stillbirth on consultant obstetrician gynaecologists. | 8 consultant obstetrician gynaecologists (50% of consultant obstetrician gynaecologists in the hospital). | Stillbirth | The lived experiences, personal feelings and professional impact. |
|  | Oe M (2018) *Burnout,* *Psychological symptoms, and secondary traumatic stress among midwives working on perinatal wards: A cross-cultural study between Japan and Switzerland.*Japan and Switzerland | Cross-sectional survey | To explore cross-cultural differences in symptoms of burnout, anxiety, depression, general psychological distress, and secondary traumatic stress between Asian (Japan) and European (Switzerland) midwives | 170 midwives; 51 from Japan and 119 from Switzerland | No definition | Secondary Traumatic Stress Scale (STSS) |
|  | Pastor Montero SM (2011)*Experiences with perinatal loss from the health professionals' perspective.*Spain | Qualitative study – individual interviews | To know the experience of health professionals in situations of perinatal death and grief and to describe their action strategies in the management of perinatal loss. | 9 nurses, 3 midwives, 5 nursing auxiliaries and 2 obstetricians from the Maternal-Infant Unit of a hospital | The experience regarding some perinatal loss situation in the professional practice. | How do you feel when you have to face these situations? What does perinatal loss mean to you? How do your beliefs about perinatal loss affect the parents’ death and grief experiences? How do you believe the parents live this experience? |
|  | Rice H (2013) *Bearing witness: midwives experiences of witnessing traumatic birth.*Australia | Qualitative study – individual interviews | To enable midwives to describe their experiences and to determine if they are at risk of negative psychological sequalae similar to those in other caring professions. | 10 currently or previously registered midwives with varying amounts of experience. | Trauma is ‘in the eye of the beholder’  Meaning that if the participant had experienced witnessing the birth as traumatic then it was traumatic. | The experiences of witnessing traumatic birth. |
|  | Robinson KA (2023) *Cross-sectional study of the frequency and severity of traumatic childbirth events and how they affect maternity care clinicians.*USA | Cross-sectional survey | To describe the frequency and severity of traumatic childbirth events (TCEs) and how they affected the professional practice and personal lives of maternity care clinicians. | RNs (n=104), certified nurse-midwives (n=17), attending physicians (n=28), and resident physicians (n=11). | Shoulder dystocia was the most frequently observed traumatic childbirth event and maternal death was the most severe traumatic childbirth event. | TCEs based on review of the literature as events that may provoke psychological distress and defensive practice behaviours, specifically, shoulder dystocia, uncontrolled postpartum haemorrhage, stillbirth/infant death, unsuccessful newborn resuscitation, maternal death, uterine rupture, and instrument injury to mother or newborn during the birth process.  The two items, influence on professional practice and on personal life, were measured independently with 5-point Likert responses (none, very mild, mild, moderate, and major). |
|  | Schrøder K (2016a) *Psychosocial health and well-being among obstetricians and midwives involved in traumatic childbirth.*Denmark | Cross-sectional survey | Investigates the self-reported psychosocial health and well-being of obstetricians and midwives in Denmark during the most recent four weeks as well as their recall of their health and well-being immediately following their exposure to a traumatic childbirth. | 293 obstetricians and 944 midwives, members of professional organizations. | A birth where the infant or mother had suffered presumed permanent, severe and possibly fatal injuries related to the birth. | Copenhagen Psychosocial Questionnaire (COPSOQII) on six scales: burnout, sleep disorders, general stress, depressive symptoms, somatic stress and cognitive stress. |
|  | Schrøder K (2016b) *Blame and guilt – a mixed methods study of obstetricians’ and midwives’ experiences and existential considerations after involvement in traumatic childbirth.*Denmark | Mixed methods study - a national survey and a individual interviews | This descriptive study investigated the numbers and proportions of obstetricians and midwives involved in such traumatic childbirth and explored their experiences with guilt, blame, shame and existential concerns. | 293 obstetricians and 944 midwives, members of professional organizations.  6 obstetricians and 8 midwives participated in interviews. | A birth where the infant or mother had suffered presumed permanent, severe and possibly fatal injuries related to the birth. | 1. I have become a better midwife or doctor due to my experiences from the traumatic birth  2. The event gave rise to personal development opportunities of an emotional and/or spiritual character  3. In the beginning I felt guilty that things turned out the way they did  4. I will always feel some sort of guilt when thinking about the event  5. Comments or behaviour from one or several colleagues caused more guilty feelings and/or lower self-esteem  6. The traumatic event has made me think more about the meaning of life  7. Memories of what happened to the patient kept troubling me for a long time after the event  8. I worried a lot about what my clinical peers would think about me after the event  9. I worried about an official complaint (or the possibility of one)  10. For a while after the event I felt shunned by some of my clinical colleagues  11. I considered moving to another institution because of the event  12. I considered leaving my profession because of the event  13. The patient and/or the next of kin blamed me for what happened |
|  | Schrøder K (2019) *Second victims in the labor ward: Are Danish midwives and obstetricians getting the support they need?*Denmark | Cross-sectional survey | To describe midwives' and obstetricians' experiences on the level of support from colleagues and managers in Danish labour wards following adverse events. | 469 midwives, 124 obstetricians | A birth where the infant or mother had suffered presumed permanent, severe, and possibly fatal injuries related to the birth. | Medically Induced Trauma Support Services (MITSS) survey  Resilience in Stressful Events Organizational Assessment Survey (RISE)  Version II of the Copenhagen Psychosocial Questionnaire (COPSOQII) |
|  | Sheen K (2015) *Exposure to traumatic perinatal experiences and posttraumatic stress symptoms in midwives: Prevalence and association with burnout.*UK | Cross-sectional survey | To investigate midwives’ experiences of traumatic perinatal events encountered whilst providing care to women, and to consider potential implications. | 421 midwives members of the Royal College of Midwives. | Criterion A of the DSM-IV-TR; that the midwife witnessed or listened to an account of an event where they perceived the mother and/or her child to be at risk of serious injury or death and where they (the midwife) experienced a sense of fear, helplessness or horror. | Impact of Event Scale-Revised (IES-R)  Maslach Burnout Inventory Human Services Survey (MBI) measuring emotional exhaustion (EE), depersonalisation (DP) and personal accomplishment (PA)  Empathic Concern (EC) subscale from the Interpersonal Reactivity Index (IRI) |
|  | Sheen K (2016a)*The experience and impact of traumatic perinatal event experiences in midwives: A qualitative investigation.*UK | Qualitative study - individual interviews | To investigate midwives’ experiences of traumatic perinatal events and to provide insights into experiences and responses reported by midwives with and without subsequent posttraumatic stress symptoms. | 35 midwives, members of the Royal College of Midwives, after participation in a survey. | Perceived the mother and/or her child to be at risk of serious injury or death and where the midwife experienced a sense of fear, helplessness or horror. | Questions on event characteristics, perceived response/impact, supportive and helpful strategies and reflections over time. |
|  | Sheen K (2016b) *What are the characteristics of perinatal events perceived to be traumatic by midwives?*UK | Qualitative study - open-ended question in survey | To investigate the characteristics of events perceived as traumatic by UK midwives. | 421 midwives members of the Royal College of Midwives. | Perceived the mother and/or her child to be at risk of serious injury or death and where the midwife experienced a sense of fear, helplessness or horror. | A short written description of a traumatic perinatal event they had witnessed, or had been recounted by a woman (‘listened to’). |
|  | Sheen P (2022) *Which events are experienced as traumatic by obstetricians and gynaecologists, and why? A qualitative analysis from a cross-sectional survey and in-depth interviews.*UK | Mixed methods - cross-sectional survey and in-depth interviews. | To explore the events perceived as traumatic by obstetricians and gynaecologists (O&G), and to examine factors contributing to the perception of trauma. | 1095 fellows, members and trainees of RCOG in the survey and 43 in-depth interviews. | Maternal or neonatal death/stillbirth, haemorrhage and events involving a difficult delivery were most frequently reported. Sudden and unpredictable events, perceived preventability, acute sensory experiences and high emotionality contributed to trauma perception. | The nature of traumatic events in this clinical setting.  . |
|  | Slade P (2018)*A programme for the prevention of post-traumatic stress disorder in midwifery (POPPY): indications of effectiveness from a feasibility study.*UK | Longitudinal study | To identify potential impacts of POPPY on midwives’ understanding of trauma, their psychological well-being and job satisfaction. | 153 midwives completed self-report questionnaires. Measures were repeated 6 months after training with POPPY. | A perinatal event where they believed themselves or someone else to be in danger of serious injury or death, and where they experienced a sense of intense fear, helplessness or horror in response. | Impact of Event Scale – Revised (IES-R)  Maslach Burnout Inventory Human Services Survey (MBI)  Attitudes to Professional Role scale |
|  | Slade P (2020)*Work-related post-traumatic stress symptoms in obstetricians and gynaecologists: findings from INDIGO, a mixed-methods study with a cross-sectional survey and in-depth interviews.*UK | Mixed methods - cross-sectional survey and individual interviews | To explore obstetricians’ and gynaecologists’ experiences of work-related traumatic events, to measure the prevalence and predictors of post-traumatic stress disorder (PTSD), any impacts on personal and professional lives, and any support needs. | 1095 fellows, members and trainees of RCOG in the survey and 43 in-depth interviews. | Criterion A1 and A2 of DSM-IV-TR; event involving actual or perceived threat to life, where the respondent appraised this with fear, helplessness or horror. | Impact of Event Scale  Revised (IES-R)  Maslach Burnout Inventory (MBI)  Sheehan Disability Scale  Interpersonal Reactivity Index (Empathic Concern subscale)  Professional Role scale  Two questions whether  specific support for obstetricians and gynaecologists following a traumatic event was needed (Yes/No) and if ‘Yes’, what participants thought would be helpful to support in dealing with workplace traumatic events. |
|  | Toohill J (2019) *Trauma and fear in Australian midwives.*Australia | Mixed methods - cross-sectional survey with closed and open-ended questions | To determine prevalence of birth related trauma and fear in midwives and associations with midwives’ confidence to advise women during pregnancy of their birth options and to provide care in labour. To describe midwives’ experiences of birth related trauma and/or fear. | 249 midwives completed the survey. 170 midwives wrote about their experiences of personal and/or professional trauma. | No definition. Personal and/or professional experience of birth as traumatic (yes/no). | Level of birth fear (1 = no fear to 10 = high fear)  ‘Practice Concerns Scale’ measuring midwives’ levels of confidence (1 = confident to 10 = not confident) and levels of worry (1 = not worried to 10 = extremely worried) around advising women of their birth options, and providing care for women in labour |
|  | Wahlberg A (2017a) *Self-reported exposure to severe events on the labour ward among Swedish midwives and obstetricians: A cross-sectional retrospective study*Sweden | Cross-sectional survey | To assess the self-reported exposure rate of severe events among midwives and obstetricians on the delivery ward and the cumulative risk by professional years and subsequent investigations and complaints. | 1459 midwives (response rate 39.9%) and 706 obstetricians (response rate 47.1%). | 1) the death of an infant due to delivery-related causes during childbirth or while on the neonatal ward; 2) an infant being severely asphyxiated or injured at delivery; 3) maternal death; 4) very severe or life threatening maternal morbidity; 5) other stressful events during delivery, such as exposure to violence or aggression. |  |
|  | Wahlberg A (2017b) *Post-traumatic stress symptoms in Swedish obstetricians and midwives after severe obstetric events: a cross-sectional retrospective survey.*Sweden | Cross-sectional survey | To examine post-traumatic stress reactions among obstetricians and midwives, experiences of support and professional consequences after severe events in the labour ward. | 1459 midwives (response rate 39.9%) and 706 obstetricians (response rate 47.1%). | The child died or was severely injured during delivery; maternal near miss; maternal mortality; and other events such as violence or threat. | Screen Questionnaire Posttraumatic Stress Disorder (SQ-PTSD), based on DSM-IV 4th edition |
|  | Wahlberg A (2019) *The erratic pathway to regaining a professional self-image after an obstetric work-related trauma: A grounded theory study.*Sweden | Qualitative study – individual interviews | To explore the process that Swedish [midwives](https://www-sciencedirect-com.docelec.u-bordeaux.fr/topics/nursing-and-health-professions/midwife) and [obstetricians](https://www-sciencedirect-com.docelec.u-bordeaux.fr/topics/nursing-and-health-professions/obstetrician) go through after a severe event in the maternity unit. | 7 midwives and 7 obstetricians who had earlier participated in a survey. | The child died or was severely injured during delivery; maternal near miss; maternal mortality; and other events such as violence or threat. | Questions regarding the participants’ experiences of received support, the medico-legal procedures, and their own strategies and actions taken. |
|  | Wahlberg A (2020) *Left alone with the emotional surge – A qualitative study of midwives’ and obstetricians’ experiences of severe events on the labour ward.*Sweden | Qualitative study – individual interviews | To explore midwives’ and obstetricians’ experiences, reactions and interpretations of being part of a severe event on the labour ward. | 7 midwives and 7 obstetricians. | The child died or was severely injured during delivery; maternal near miss; maternal mortality; and other events such as violence or threat. | Questions about experiences of one or several severe events; event-characteristics, perceived response, and support and/or strategies that had been helpful. |
|  | Wallbank S (2013) *Predictors of staff distress in response to professionally experienced miscarriage, stillbirth and neonatal loss: A questionnaire survey.*UK | Cross-sectional survey | To explore the extent of staff distress, and its predictive factors, in a sample of United Kingdom nursing and medical staff. | 38 doctors, 42 nurses, 104 midwives employed in obstetrics and gynaecology settings. | Experience of loss in a professional capacity (via miscarriage, neonatal death or stillbirth). | Impact of Events Scale  (IES)  Positive And Negative Affect Scale (PANAS)  Brief COPE  Work Environment Scale  (WES) |
|  | Walker AL (2020) *Impact of traumatic birth on Australian obstetricians: A pilot feasibility study.*Australia | Mixed method: cross-sectional survey and individual interviews | To assess the feasibility of conducting a binational survey of Australia obstetricians, trainees, and general practitioner obstetricians, to determine the prevalence of trauma exposure and associated factors. | 32 obstetricians completed the survey and 8 the interviews. | Traumatic event exposure lists based upon published studies. Birth‐related trauma included exposure to maternal death or near miss, and severe intrapartum injury to baby.  Professional events included medicolegal claims, and being reported to regulatory authorities. | The Posttraumatic Diagnostic Scale for Diagnostic and Statistical Manual of Mental Disorders–5 (PDS–5)  The Copenhagen Burnout Inventory‐subscale Work (CBI‐W)  The Posttraumatic Growth Inventory short form (PTGI‐SF) |
