# Supplementary material for: The impact of severe perinatal events on maternity care providers: a scoping review
Source: BMC Health Serv Res. 2024 Feb 7;24:171. doi: 10.1186/s12913-024-10595-y (PMC10848539; doi:10.1186/s12913-024-10595-y)
Supplement: Supplementary file 4 — Additional file 4. Questions for the content analysis. [file 12913_2024_10595_MOESM4_ESM.docx]

Questions for the content analysis

Aim of our scoping review: To explore and synthesize what is known in the literature about the impact of traumatic events during the perinatal period on care providers and how these experiences affect their professional practice (including their interaction with women).

Exclude papers in which traumatic events are directly linked to COVID(-measures).

|  | first author | providers | event | impact | changes | support | patterns |  |
| --- | --- | --- | --- | --- | --- | --- | --- | --- |
| # | First author (year)  Incident of traumatic events among professionals reported | Which healthcare providers have been studied? | How was the event described or articulated / by the care providers?   - Related to mother and infant outcome - Interaction   - between professionals   - between family and professionals   - Part of it or on the side line - Care context - The crucial element that made it a traumatic event - Others | What impact (emotions, responses, behaviours, etc.) did the event have on the care provider involved/studied?   - Professionally   - Relation with colleagues   - Short term   - Long term   - Litigation - Personally   - Their own mental health   - Their family | What changes in practice were associated with the event?   - In the way care is provided - In the interaction with women   In the professional role | What support was experienced?   - Formal - Informal - None | What patterns seem to appear, e.g. with regard to professional background? | Any other remarkable findings that fits the aim of our scoping review |
|  | Amir Z (2020)  Over 90% of respondents (response rate 55%) experienced exposure to a traumatic event in work in the previous year, with 58% reporting a frequency of monthly or greater. | Midwives | Events for which the respondents ‘did not feel adequately prepared’ or that they ‘found upsetting or overwhelming’. | No significant relationship was demonstrated between frequency of trauma and burnout.  The extent of distress experienced was positively related to burnout in each domain (R2 = 0.18, 0.15 and 0.09, respectively, P < 0.01).  A significant negative correlation was seen between work-related burnout score and years of work experience (P < 0.01). | Not mentioned | Not mentioned | Burnout is common among midwives. Exposure to discrete traumatic perinatal events experienced by women under their care contributes to this. |  |
|  | Baas MAM (2018)  The response rate was 42.8%, 12.6% of the respondents had experienced a work-related traumatic event. | Obstetricians | Work-related adverse events.  Work-related traumatic event was defined as respondent meeting PTSD-criterion A according to DSM-IV (2 questions) with the event at least more than four weeks ago.  Respondents reported as high emotional impact stressors: missing a diagnosis (64.3%), doubting a medical decision (44.5%), life-threatening moments (43.2%), death of a patient (37.6%), feeling they could not help the patient (24.3%), bad news conversations  (16.1%). Other stressors reported (23.1%) included severe complications, conflicts with colleague, patient and disciplinary board complaints, discontented patients and patient aggression or violence. | 11.8% met the criteria for current posttraumatic stress disorder.  Among the respondents, 5.1% increased their use of alcohol, drugs and/or nicotine and 1.5% used medication they normally would not use. | 33.7% have at some point considered leaving their medical profession. The most common reasons were a high workload, varying shifts, high responsibility, work/life imbalance, conflicts with colleagues, new interests and work culture related problems. The group that had considered leaving the profession had a significantly higher PTSD prevalence rate (0.7% vs 3.0%, p = 0.01).  0.6% gave up practicing as an ObGyn because of emotional stressors.  One in two ObGyns (55.2%) of the respondents became more defensive in their decision making and 24.4% changed their work habits as a result of adverse events (e.g. no longer doing nightshifts, not performing surgery alone or no longer performing vaginal breech deliveries). | Of all responders, 60.0% thought the current support services after adverse events are insufficient. 12% reported to have a support protocol or strategy in their hospital after adverse events. The most common strategies to cope with emotional events were to seek support from colleagues (87.4%), to seek support from family or friends (72.2%), to discuss the case in a complication meeting or audit (42.6%) and to find distraction. 82% would prefer peer-support with direct colleagues after an adverse event.  53% reported never having formally learned, 22.5% during a peer support group, 10.5% during specialist training, 4.8% during medical school, 4.8% during additional specialist courses and 33.8% learned through other ways. With the statement that there is room to express emotions on the ward or within their department after experiencing an emotional event, 80.8% agreed. | Work-related events can be traumatic and subsequently can lead to posttraumatic stress disorder.  Often there is no standardized support after adverse events. Most obstetricians prefer peer-support with direct colleagues after an adverse event. | More awareness must be created during medical training and organized support must be implemented. |
|  | Beck CT (2012) | Labor and delivery nurses | Traumatic birth as identified by the participant.  Directly experiencing a stressor, which can involve actual or threatened death or serious injury or indirectly experiencing by witnessing it occurring to another individual or by learning about it.  Nurses frequently used phrases such as “the physician violated her,” “a perfect delivery turned violent,” “unnecessary roughness with her perineum,” “felt like an accomplice to a crime.” | 35% of the respondents reported moderate to severe levels of secondary traumatic stress. 26% met all the diagnostic criteria for screening positive for PTSD due to exposure to their patients who are traumatized.  The list seemed endless of the distressing emotions nurses experienced during traumatic births.  Complicating L&D nurses’ helplessness was the guilt that ensued when at times nurses felt like they failed their patients when they did not speak up and challenge/question the obstetricians’ practices. | Traumatic births led some nurses to consider leaving L&D. Some nurses moved from direct patient care to managerial positions, graduate school, academia, or to the antepartum area, that was a much less intensive setting. The threat of litigation  in traumatic births also played a part in nurses’ considering leaving L&D. | For some nurses prayer was an integral part of getting through and coping afterwards regarding traumatic births.  Once traumatic births are over, informal and formal debriefing helped nurses. Informally nurses, and at times with physicians and anesthesia staff, came together to debrief.  Formal debriefing sessions with a chaplain or social services were also scheduled after a bad outcome. Sharing their thoughts and feelings with other staff and not keeping them bottled up inside helped to defuse exposure to a difficult birth.  Also helpful for some nurses was visiting with the patients afterwards to discuss the births.  Some hospitals had team training exercises using  simulation to practice emergency experiences. |  |  |
|  | Beck CT (2015) | Midwives | Traumatic birth as identified by the participant.  The top 3 types of traumatic births described by the CNMs were fetal demise/neonatal death, shoulder dystocia, and infant resuscitation. | 29% of the CNMs reported high to severe STS, and 36% screened positive for the Diagnostic and Statistical Manual of Mental Disorders, Fourth Edition diagnostic criteria for PTSD due to attending traumatic births.  Respondents felt a sense of powerlessness from not being able to protect their patients from this traumatizing experience. Other midwives vividly described their terrifying helplessness.  The smallest details of traumatic births haunted midwives as they relived every aspect over and over again in their minds. Reoccurring nightmares are very much a part of attending traumatic births.  Sadness, anxiety, anger, fear, guilt, and numbness were distressing emotions that exemplified the experiences of attending traumatic births. | Often, midwives shared that their belief in the birth process was shaken. Some CNMs became more guarded and cautious in their practice after attending traumatic births, whereas other midwives described how their “index of suspicion” during future births was heightened due to their experiences.  A pervading sense of fear and a sense of intense doom as the birth got closer were also experienced by CNMs. For some CNMs, their traumatic childbirth experiences led them to not only contemplate leaving midwifery but actually to make some definite changes in their careers, like going into academia, administration or staff nursing.  Approach to practice changed, such as being quicker to intervene, more assertive, more vigilant, more conservative and more fearful. | In CNMs’ narratives about the traumatic births that they had attended, they would often share that what helped them was the support of the entire obstetric team: fellow CNMs, obstetricians, nurses, and neonatologists.  Not all CNMs, however, were fortunate to be encompassed by all this support. “Abandoned” and “betrayed” after a traumatic birth were adjectives used to describe how some CNMs felt as a result of not being supported by other members of the obstetric team. Unsupportive physicians led some CNMs to report that they worked in a hostile environment that was toxic and unsafe and in which an unwelcome relationship existed with the collaborating physicians. |  |  |
|  | Beck CT (2016) | Labor and delivery nurses | Traumatic birth as identified by the participant. | The mean sum total score of the Posttraumatic Growth  Inventory (PTGI)  for the labor and delivery nurses was 58.53, which indicated that labor and delivery nurses who had cared for women during traumatic births reported vicarious  posttraumatic growth near a moderate degree.  The total mean sum score for the Core Beliefs Inventory (CBI) was 21.85, which is in the midpoint of the inventory’s possible range of 0 to 45, indicating labor and delivery nurses who cared for women during traumatic births reported near a moderate degree of disruption in their assumptive world.  Appreciation of Life was the dimension of the Posttraumatic Growth Inventory that reflected the highest growth.  The most frequently cited shift in priorities in nurses’ lives focused on higher valuation of their families and friends, especially their children. | The development of more compassion for the families who experienced traumatic births was described by labor and delivery nurses most frequently. Positive changes from traumatic births included the increased closeness with co-workers that developed as labor and delivery nurses learned they could count on their colleagues during a difficult birth.  Labor and delivery nurses explained that these difficult births made them stronger and more capable than they ever knew. An area of positive change for some labor and delivery nurses, which they believed was directly tied to traumatic births, was in the development of more assertiveness as a patient advocate.  As a result of their care for families who had experienced perinatal loss, another new path labor and delivery nurses were drawn to was perinatal bereavement. Nurses also took on active roles in mentoring new labor and delivery nurses to always be prepared for any bad outcomes and to rejoice in good outcomes. |  |  |  |
|  | Beck CT (2016) | Midwives | Traumatic birth as identified by the participant. | CNMs reported a small degree of both disruption of their core beliefs and vicarious posttraumatic growth. Personal Strength was the dimension of the  Posttraumatic Growth Inventory that reflected the highest growth. | The traumatic births, helped to realize how resilient they were.  Attending traumatic births also taught CNMs how to become more compassionate and empathetic toward other providers and patients. |  |  |  |
|  | Becker J (2023) | Midwives | Very early neonatal death | Midwives reported deep sadness leading to internal struggles. Use of spirituality as ‘God’s plan’ as an explanation. Participants articulated that they concentrated on active solutions to save babies during labour, such as vigilant foetal heart rate monitoring and partogram. | Building resilience through education of midwives and women to provide a woman-centred approach to care. | Not mentioned |  |  |
|  | Ben-Ezra M (2014)  The response rate was 39%. A total of 59.3% (n=16) of the nurses reported being exposed to a traumatic event  according to the Diagnostic and Statistical Manual of Mental Disorders, Fourth Edition (DSM IV) criterion A for posttraumatic stress disorder (PTSD) excluding perinatal death. | Obstetrics nurses | Perinatal death | Higher level of psychiatric symptoms [posttraumatic stress disorder (PTSD), depressive, and psychosomatic symptoms] at time 2 (after exposure to perinatal death) in comparison to time 1. The results showed a higher level of psychiatric symptoms (PTSD, depressive, and psychosomatic symptoms) in the exposed group in comparison to the non-exposed group. | Not mentioned | Not mentioned |  |  |
|  | Calvert I (2015) | Midwives | Self-identified as having experienced a traumatic practice experience.  Neonatal and maternal deaths, preeclampsia, breech birth, resuscitation, postpartum hemorrhage, amniotic fluid embolus, human rights issues, insufficient staffing, and the power of the medical team influencing the outcome for the woman and her baby.  Violence - The midwives’ stories illustrate that symbolic violence is a part of the institutional culture of midwifery in New Zealand and comprises rumor, gossip, and negative nonverbal communications. Violence or bullying from women, other midwives, management, or medical staff was present in all but 1 of the 16 stories told by the participating midwives.  In the midwives’ narratives, the conflict stemmed from the cultural and  historical ideas present in the philosophical underpinnings of the medical and midwifery models of care, which influence the ideas of each profession as Judy explains, If your practice is seen to be outside what they consider normal, practicing in the midwifery model is not seen to be OK. | The event affected their personal and professional identities creating biographical disruption thus increasing the physiological and psychological symptoms associated with the traumatic event. Loss of identity created grief. | Practice changes identified by the participants in the study involved midwives giving up midwifery, refusing to work across the scope or alter the way they practice. | The study highlighted the emotional needs of the midwife were not considered by management; therefore, psychological debriefing or professional supervision was not available unless in a management position or following maternal death.  The consensus was that risk management debriefing on its own, although important for management, does not provide the emotional support required by the midwives following a traumatic practice experience.  Although the importance of psychological debriefing from a mental health worker, when the midwife was ready, was acknowledged, professional supervision from someone with knowledge of midwifery, preferably from outside of the organization, was viewed as paramount to lessening the trauma. |  |  |
|  | Çankaya S (2020)  No overall incident rate was given. Response rate was 77.7% (266 of 342 potential nurses/ midwives).  About half witnessed a difficult birth  (n=124, 48.1%),  34.9% attended one, 41.5% (n=107) both witnessed and attended the loss of mother/baby during birth,  33.7% (n=87) both witnessed and attended a serious perinatal/postnatal injury experienced by the mother/ baby, about half (n=129, 50%) attended the birth of a baby with an abnormality, about a quarter (n=59, 22.9%) attended a stillbirth with dismembering  (n=72, 27.9%) maltreatment of the women by the attending physician during birth, 36.8% both witnessed and attended a birth where the requests of the woman were ignored. | Maternity nurses/ midwives | A traumatic perinatal event experience was defined according to DSM-IV-TR Criterion A (APA, 2000).  An event where they perceived that the mother and/or her baby were at risk of serious injury or death and experienced fear, helplessness, or horror in last week. | 37.2% met the criteria for posttraumatic stress disorder (% 95 CI [31.6, 43.2]). Previous birth-related traumatic experiences, transferring to another unit, and negative cognitions about the self, increase the risk of development of posttraumatic stress disorder by 63% (F=7.638, P < 0.001).  A positive correlation was found between the outcome variable PTS total score and the number of years in the profession, the number of traumatizing events, quality of work life, burnout symptoms, compassion fatigue, posttraumatic cognition, negative cognition about the world, and negative cognition about the self. It was found that those maternity nurses/midwives who were not happy with their profession, who witnessed a traumatic incident, who transferred to another unit, who considered quitting the profession, who had a history of a psychological disorder, and those lacked social life were more sensitive to posttraumatic stress symptoms. | Not mentioned | Not mentioned |  |  |
|  | Çankaya S (2021) | Midwives | Participants were asked to describe one traumatic birth experience. 15 midwives had experienced trauma due to difficult labour (shoulder dystocia, occiput posterior presentation, precipitated labour, prolonged labour, third/fourth degree perineal tear, vaginal variceal haemorrhage, and so on). The trauma was due to infant death in 10 cases and maternal death in four events. Midwives who witnessed maternal and infant death stated that they were affected by this event than those who witnessed difficult births. However, all the midwives who witnessed mother-infant death and difficult births stated that they were affected by this situation. | Midwives experienced highly emotional exhaustion. Two third of the midwives needed psychological support.  Midwives described many problems such as sadness, repeated flashbacks, guilt and responsibility, fear, empathy, anxiety, difficulty in informing the family, accusation by others. Due to the fact that they had intensive workload and they were not allowed to take some time off the work, they were unable to feel the pain about the event. | They performed an increasingly defensive practice. While some of these changes were right practices (midwives began to  take women's medical history in detail, inform the doctor in risky situations, give training to pregnant women, and so on), others did not overlap with the evidence-based midwifery practices (episiotomy practice for every pregnant woman, oxygen initiation immediately after the birth even if there is no need, and so on). Midwives took on a more defensive role to take precautions and reduce the complaint about the malpractice claim owing to the negative experience they had lived before. | Midwives were not prepared enough for traumatic events and that most traumatic births were simply ignored in their workplace. Eventually, it was determined that midwives received support mostly from their colleagues in case of a traumatic birth. |  |  |
|  | Cauldwell M (2015) | Midwives, doctors in training (trainees) and consultant obstetricians | Maternal death | Maternal death has a major impact on professionals’ feelings of grief, guilt and shame, which they are reluctant to talk about. Guilt was perceived as a long-lasting emotion, often remaining unresolved and leading to feeling inadequate. | Not mentioned | The maternity department had a system for “debriefing” meetings for staff (facilitated by an obstetrician and a psychologist), held within days of a serious incident (i.e. unexpected stillbirth, severe morbidity or maternal death). Most felt “debriefing” was an appropriate arena to explore some of the issues raised by the maternal death. Consultants and one senior midwife spoke about inadequate debriefing that was not universally beneficial.  All felt they would seek support from colleagues when confronted with maternal death. Maternity professionals expressed a desire for training to prepare themselves to respond effectively in the event of maternal death. |  |  |
|  | Cohen R (2017)  A response rate of 45.8%. 94.6% experience a traumatic births at work, 75.2% experienced  1–30 traumatic births at work, and 13.9% reported over 30 traumatic births. | Midwives | Participants were asked to report if they were exposed to traumatic births during their job.  Five main domains: 1-death of baby/mother; 2-medical complications; 3- aggression and violence towards the nurse-midwife; 4-job accidents and inexperienced medical staff; 5-emergency deliveries, life-endangering situations and 6-other, which included experiences that could not be categorized into any of the above classes.  11.6% experienced aggressive behavior from the mother or her family.  Job accidents or lack of professionalism of the medical staff  (8.2%). The woman suffered from pre-eclampsia and the medical stuff lacked experience.  Other 5.8% The woman was devastated because she couldn't go through natural birth as she hoped. | Results indicated relatively high levels of Compassion Satisfaction, which may mitigate, at least to some degree, the negative aspects of Compassion Fatigue. PTSD levels were significantly and positively correlated with Secondary Traumatic Stress and Burnout. Sixteen per cent presented with PTSD symptoms of clinical significance. Also, seniority was significantly and positively correlated with burnout and PTSD symptoms. | Not mentioned | Not mentioned |  |  |
|  | Farrow VA (2013) | Obstetricians | Stillbirth | Overall, grief was the most common emotional response to a stillbirth, with 96.6% of the respondents indicating they ‘‘very much’’ (53.7%) or ‘‘somewhat’’ experienced grief. Other common reactions that obstetricians ‘‘somewhat’’ experienced were self-doubt (56.6%), fear of legal action (55.5%), feelings of guilt (53.6%) and self-blame (48.0%). More than a third of the sample (38.2%) ‘‘somewhat’’ experienced depression. Most respondents did not (‘‘not at all’’) experience an inability to sleep (79.9%), or PTSD1 (82.7%).  Significant psychological impact on the obstetrician was associated with older age, solo practice, higher volume practices and higher proportion of Medicaid patients; gender was not found to be associated with psychological impact when controlling for age. | Not mentioned | Further, greater self-reported performance and training regarding maternal and family counseling, management of stillbirth, and knowledge of stillbirth evaluation was associated with greater levels of grief. |  |  |
|  | Favrod C (2018) | Midwives and NICU nurses | Work-related stressors were classified into five themes: “Working environment,” “Nursing/midwifery care,” “Dealing with death and dying,” “Case management” and “Others.” Forty-six (46.3%) percent of these were classified as traumatic work-related stressors. Categorizing these 639 work-related stressor examples into traumatic vs. non-traumatic work-related stressors (according to DSM 5) resulted in 296 (46.3%) traumatic work-related stressors.  “Working environment” included 205 situations (32.1%) in total. These situations were related to the organization of work and relations with medical staff (physicians, co-workers, workers from other wards, as well as superiors) or were seen as consequences of a lack of work-organization or poor relationships among staff, e.g., “Work overload and lack of personnel.” | 10.1% of midwives scored high or severe on STSS levels. When controlling for socio-demographic variables, NICU nurses had a higher STSS total score and higher STSS subscales scores and less HADS anxiety subscale scores than hospital midwives did. Group comparisons regarding the total number of traumatic vs. non-traumatic stressors showed no significant difference between professional groups [­2(1) = 0.655; p = 0.418]. | Not mentioned | Measures, such as teaching strategies to amend the subjective appraisal of the traumatic stressors or providing time to recover in-between frequently occurring work-related traumatic stressors might not only improve the mental health of professionals but also decrease sick leave and improve the quality of patient care. |  |  |
|  | Fontein Y (2018) | Midwives | Various work-related traumatic events: witnessing birth trauma/complications (34%), death (28.3%), (mis) management of care (19.8%), events related to the perceived social norm of maternity services’ practitioners (9.5%), events related to environmental and contextual issues (5.6%) and to (mis) communication (2.8%)  (Intra-uterine) death 28 (26.4)  Resuscitation — including maternal resuscitation 11 (10.4)  Shoulder dystocia 9 (8.5)  Suboptimal — (mis) management of care 8 (7.5)  Severe maternal morbidity 7 (6.6)  Lack of collegiality — including bullying 6 (5.7)  Delayed assistance (help) 6 (5.7)  Postpartum haemorrhage 4 (3.8)  Midwife’s care (management) being questioned by other  practitioners  4 (3.8)  Risky — unsafe management of care (own and other’s) 4 (3.8)  Witnessing very invasive (inhumane) ways of performing  interventions  3 (2.8)  Insolvable maternal despair — including maternal inability to cope emotionally  3 (2.8)  Involvement in legislation 3 (2.8)  Suboptimal communication with hospital staff 3 (2.8)  Maternal death 2 (1.9)  Woman with a complicated medical history combined with negative birth outcome 2 (1.9)  Unsafe environment — including violence (aggression) 2 (1.9)  Being pregnant combined with involvement in neonatal mortality 1 (0.9) | In all, 74.5% of the participants still experienced the influence of work-related events in day-to-day practice and 37.5% still experienced the effects in their personal life. The scores of three participants (3.2%) indicated the likelihood of post-traumatic stress.. However, most responses had a rather negative character, represented in reports of being less at ease and reduced happiness at work, including reduced work satisfaction. | The influence experienced on day-to-day practice showed a mean ± SD score of 2.2 ±0.96 (range 1–5). The participants described if and how they were still aware of the influence of the described upsetting, distressing or traumatic work-related event on work floor level, their day-to-day practice. A quarter of the respondents reported to experience no after-effects of the event. Three-quarters reported positive and negative experiences because of the event. Positive influences were identified as professional development or taking more time for women. The events also resulted in change of care management, anticipating worst-case scenarios. Leaving midwifery was also reported | Sharing the experience with colleagues, family and friends, a supervisor or the woman involved in the event, was the most common response. |  |  |
|  | Goldbrot J (2011) | Nurses | Events in birth identified as traumatic by the participants. | All the participants shared symptomology of STSD. | Not mentioned | Not mentioned |  |  |
|  | Halperin O (2011) | Midwives | An extremely stressful situation they had experienced. The main life-threatening situations described by the participants were the infant’s death, the mother’s death, a third or fourth degree of perineal tear, hysterectomy, shoulder dystocia, and/or other complicated births. | The participants described an array of emotional reactions to their involvement in extremely stressful situations.  The tragic consequences for the mothers and/or newborns resulted in profound feelings of sadness, helplessness, and loss for the participants as well. These tragic situations challenged their sense of professional and personal competency, making them feel alone, emotionally overwhelmed, and like failures. The participants also described intense physical symptoms.  The intensity of these experiences often extended beyond their immediate impact, with long-lasting effects that were difficult for the participants to overcome. | Professionally, I lost my confidence; I did not want to do births anymore. | Colleagues’ reactions played a significant role in shaping the participants’ experience of the stressful event. Whereas some colleagues were supportive, others blamed them for the tragic consequences and even mocked their behaviors, rather than helping them to reconstruct their shattered sense of professional self-reliance. Consequently, the participants experienced intense feelings of exclusion, rejection, and loneliness, and their unabated distress interfered with their continued ability to function professionally. Some participants recalled receiving support from the supervisory staff, whereas others reported no show of support whatsoever. Whereas receiving support served to empower some participants, the lack or total absence of support received by others added to their profound experience of loneliness, abandonment, and overall trauma. | Stressful childbirth situations can have a long-term impact on midwives’ professional and personal identities. Midwives need to feel supported and valued in order to deal with emotional stress. Incorporating clinical supervision by experienced midwives can serve as a supportive framework for other midwives. |  |
|  | Hildingsson I (2013) | Midwives | Experience of a critical situation. |  | 169 of 475 midwives considered leaving the profession, 15.4% mentioned the experience of a critical event as a reason. | Not mentioned |  |  |
|  | Hutti MH (2016) | Nurses working in obstetric , surgery and emergency departments | Fetal loss | Nurses had positive and negative feelings associated with caring for women after fetal loss. The negative feelings reported by nurses mirror some emotions commonly associated with compassion fatigue, such as feeling overwhelmed and incompetent, use of alcohol to relax after work, and recalling situations that they will never forget. | Not mentioned | Not mentioned |  |  |
|  | Javid N (2019) | Midwives | Experience in caring for women with undiagnosed casa praevia during labour and birth. Further experience of involvement in neonatal death or near miss due to vasa praevia. | Expressed feelings of shock and horror witnessing the (potentially) dying baby as well as guilt and anger and ruminative thoughts after the event. Devastating and dreadful experience (dreadful, destroying, very traumatic, very stressful, terrible, catastrophic and disaster). Personal impacts like feeling scared, being chocked and feeling guilty. | Not mentioned | Most midwives had some kind of professional support and debriefing from their colleagues and hospitals.  One midwife, however, who was involved in a neonatal death, reported that ‘the manager said to go home and that’s it . . . I never had debriefing for that case’. |  | The narratives highlight the challenges in maternity care providers, pointing high importance to the need for antenatal diagnosis of vasa praevia to improve perinatal outcomes. |
|  | Jonas-Simpson CF (2013) | Obstetrical and neonatal nurses | Perinatal death | Nurses’ grief is significant. Participants discussed how growth emerged through the difficulty, challenges, and anguish of grieving while caring for families with a perinatal loss. Although experiencing grief while with families who experienced a perinatal loss was described as being “hard” and “difficult,” or as “an emptiness,” and “a big black hole sometimes,” it was also viewed as a rewarding experience that inspired change and growth. Participants described how grief had an impact on them personally and professionally. We chose not to separate the professional from the personal, as these coexist in life. |  | Support from colleagues was very significant in helping them to find their way through grief. Most often, the meaningful support came from fellow nurses and when it was not available, it was clearly missed, as one participant stated. While the nurses’ family members tried to help, it was their colleagues who understood their experience of grief and provided the most support. | Nurses who grieve require acknowledgment, support, and education. Supporting staff through their grief may ultimately have a positive impact on quality of work life and home life for nurses and quality of care for bereaved families. |  |
|  | Jones K (2015) | Midwives | Stillbirth | Each midwife experienced shock and an intense and personal sense of loss. This created tension as they strived to cope with their own emotions and continued to care for the woman and her family. | Not mentioned | Not mentioned |  |  |
|  | Katsantoni K (2019) | Assistant nurses,  registered nurses and  midwives | Caring for a woman who experienced a traumatic birth experience. | The majority of participants were at the high-risk category for CF/STS (73.9%) while only 19.8% and 5% of nurses expressed high potential for CS and BO, respectively. Those who cared for a woman with a traumatic labour experience reported higher levels of compassion fatigue compared to those who did not, and higher levels of secondary post-traumatic anxiety than those who did not. | Not mentioned | Not mentioned |  | The authors suggested developing educational and supportive interventions to prepare maternity professionals to cope with the emotional content of their work in the face of trauma. |
|  | Kave YV (2023) | Midwives | Caring for women with perinatal loss | Participants experienced feelings of guilt, stress and depression. | Not mentioned | Participants relied on their own coping mechanisms and then appreciated being able to debrief with peers but many relied heavily on family especially when no peer-debriefing was available. |  |  |
|  | Kerkman T (2019)  The response rate was 23%, with 691 questionnaires eligible for analysis. 13% of respondents reported having experienced at least one work-related traumatic event. | Midwives | A traumatic event as identified by the participants. Most emotionally stressful events were Missing a diagnosis, death of mother or child, life-threatening moments, Doubting a medical decision, Feelings of helplessness, Delivering bad news. | 17% screened positive for PTSD, revealing an estimated PTSD prevalence of 2% among Dutch midwives. Clinically relevant anxiety symptoms were reported by 14% of the respondents, significantly more often among midwives working in primary care (P = .014). Depressive symptoms were reported by 7% of the respondents. | A significantly higher proportion of midwives in primary care reported adjusting their working conditions after being involved in a traumatic event compared with their hospital counterparts (35% vs 22%; P = .004). | The desired strategies to cope with an adverse event were peer support by direct colleagues (79%), professional support from a coach or psychologist (30%), multidisciplinary peer support (28%), and support from midwives who are not direct co-workers (17%).  Midwives who were aware of the existence of guidelines for support after an adverse event were significantly (P <.001) more satisfied about the support offered compared with midwives without guidelines regarding support. Next to this, significantly more midwives working in a hospital setting reported being aware of guidelines regarding support compared with primary care midwives. |  |  |
|  | Komachi MH (2012)  Of the 176 nurses who returned the questionnaires  (52.1% response rate), 159 (90.3%) participants reported encountering a traumatic event while providing nursing care. | Nurses | Cared for a pregnant woman or a woman in childbirth who was in serious condition.  Cared for a pregnant woman or a woman in childbirth who died.  Cared for a newborn or infant in serious condition.  Cared for a pregnant woman who had an abortion or miscarriage after 6 months of pregnancy. | The highest mean trauma severity scores were associated with ‘Care for pregnant woman who had an abortion or miscarriage after 6 months of pregnancy’ (10, range 10–10) and ‘Care for newborn or infant in serious condition’ (8.5, range 7–10). The former was experienced by two nurses (1.3%) and the latter by 12 nurses (7.5%). | Not mentioned | Not mentioned |  |  |
|  | Leinweber J (2017a)  With a response rate of 15%, a 100% of the participating midwives described features of a witnessed index trauma. More than two-thirds of midwives (67.2%) reported having witnessed a traumatic birth event that included interpersonal care-related trauma features. | Midwives | Participants were invited to identify a traumatic birth event they had witnessed when providing care for a woman (the ‘index’ trauma).  The Traumatic Events in Perinatal Care List (TEPCL) included (1) death (maternal or foetal, actual or threat of); (2) injury (maternal or foetal, actual or threat of). Interpersonal birth trauma included (1) abusive care (or management); (2) poor care (e.g., witnessing or participating in a procedure that is not in the woman’s and/or the baby’s best interest); (3) interpersonal disrespect (e.g., witnessing the woman’s dignity being ignored, her wishes overridden). | Midwives recalled strong emotions during or shortly after witnessing the traumatic birth event, such as feelings of horror (74.8%) and guilt (65.3%) about what happened to the woman. Midwives who witnessed birth trauma that included care-related features were significantly more likely to recall peritraumatic distress including feelings of horror (OR = 3.89, 95% CI [2.71, 5.59]) and guilt (OR = 1.90, 95% CI [1.36, 2.65]) than midwives who witnessed non-interpersonal birth trauma. 17% of midwives met criteria for probable posttraumatic stress disorder  (95% CI [14.2, 20.0]). Witnessing abusive care was associated with more severe posttraumatic stress than other types of trauma. | Not mentioned | Not mentioned | Midwives witness traumatic birth events and may be at risk for developing posttraumatic stress symptoms. Evidence that witnessing care-related interpersonal birth trauma provokes strong emotional reactions in midwives and may have long-term implications for their psychological well- being. |  |
|  | Leinweber J (2017b) | Midwives | Trauma in childbirth is "in the eye of the beholder”, which means that if you recall an experience/event around a labour and birth as traumatic, then it was traumatic.’ Using the Traumatic Events In Perinatal Care List (TEPCL) (Leinweber et al., 2016) five categories of witnessed traumatic birth event features were listed (1) death or (2) severe injury of mother or baby (3) abusive care (4) involvement in suboptimal care and (5) disrespect of women's dignity. | Peritraumatic feelings of guilt, and a personal traumatic birth experience were strongly associated with probable Posttraumatic Stress Disorder.  A peritraumatic reaction of horror. | The intention to leave the profession | Not mentioned |  | Posttraumatic stress may contribute to attrition in midwifery. Trauma-informed care and practice may reduce the incidence of traumatic birth. |
|  | Marguiles SL (2020) | Labor and delivery, postpartum, and neonatal intensive care staff | A maternal or perinatal/neonatal loss | Three (12.5%) of the residents screened positive for PTSD symptoms; eight were positive for depression; three were positive for both. Two sought mental health treatment, and two used substances. Non-physicians, those using substances, those considering career change, and those seeking mental health treatment are more likely to experience anxiety/depression and post-traumatic stress symptoms after a maternal or perinatal/neonatal loss. | One resident (who did not screen positive for PTSD symptoms or depression) considered changing their career. 20 out 105 considered a career change. | Most (83%) residents agreed that co-workers were supportive. Most (62%) agreed that the people in charge were supportive; 12.5% disagreed. |  | Individuals with symptoms should be identified and offered additional support. |
|  | Minooee S (2021a) | Midwives | Shoulder dystocia complicated births | Fear, anxiety and doubt about their professional competence were the most common feelings experienced by midwives after SD. | For many, the first exposure to SD left them contemplating their previous attitude towards normal birth. Disturbed orientation of normal birth shifted midwives towards hypervigilance in practice. | Not having effective relationships with women and receiving poor support from colleagues were perceived to worsen the traumatic experience, whereas working in a midwifery continuity of care model and the sense of being appreciated improved midwives’ experience after the trauma. |  |  |
|  | Minooee S (2021b) | Midwives | Shoulder dystocia complicated births |  | Experience of SD was described as a dreadful event in midwives’ career. How- ever, on reflection, this experience was considered as a benefit which developed midwives’ clinical expertise and contributed to their professional empowerment. | Having faith in birth normality, support from workplace, seniority/ years of experience and self-confidence were among the factors which helped midwives in overcoming the stress after SD. |  |  |
|  | Muliira RS (2015a) | Midwives. | Witnessing a maternal death. | The results from the Death Distress Scale showed that the majority of midwives who had witnessed a maternal death had moderate to high death anxiety (93%), mild to moderate death obsession (71%) and mild death depression (53%). | Not mentioned | Most midwives coped with their distress using methods such as active coping, venting, positive reframing, self-distraction and planning. |  | There is a need for midwifery practice settings to provide respite care, education on coping with death experiences and counselling after traumatic experiences in order to maintain the well-being of midwives. |
|  | Muliira RS (2015b) | Midwives | Maternal death situations | Most participants (74.6 %) had moderate or high death anxiety. The predictors of death anxiety were: having witnessed two and more maternal death in the past 2 years [odds ratio (OR) = 3.175; p < .01]; being in charge of four or more maternal deaths (OR = 5.13; p < .01); lack of professional training in handling death situations (OR = 3.32; p < .01); and coping with maternal death situations using methods such as: planning (OR = 4.90; p < .01), active coping (OR = 3.43; p < .05) and acceptance (OR = 2.99; p B .05). | Not mentioned | Not mentioned |  | Employers need to provide deliberate support to enable midwives to cope effectively with death anxiety at work. |
|  | Nicholls EM (2021)  Response rate was 50%. The majority of respondents (84.7%) reported witnessing a traumatic birth. | Labor and delivery nurses | Participants were asked to rank the extent to which they perceived several birth situations to be traumatic on a scale from 0 (not traumatic) to 100 (most traumatic). The traumatic birth situations  were amniotic fluid embolism, birth with physical fetal anomalies, emergency cesarean, unanticipated hysterectomy following birth, inevitable loss, unsuccessful instrumental  delivery with vacuum or forceps, maternal code, maternal death, neonatal code, neonatal death, postpartum  hemorrhage, prolapsed umbilical cord, shoulder dystocia.  Participants identified maternal deaths, neonatal deaths, and maternal codes as the top three most traumatic events experienced. | 35% of respondents meeting symptom severity scores associated with STS. | STSS Scores ≥ 38 were significantly correlated with nurses considering leaving their jobs, calling out sick, or requesting an assignment change after witnessing a traumatic birth (p < 0.001). | After witnessing a traumatic birth, respondents used co-workers, family, and friends as sources of support. |  |  |
|  | Nightingale S (2018)  The actual response rate for midwives who met the inclusion criteria is uncertain. | Midwives | During the last 10 years witnessed a perinatal event that included actual or threatened death or serious injury to the mother and/or child, experienced fear, helplessness or horror.  Midwives had experienced an average of five traumatic perinatal events (mean = 4.64, SD 3.49) throughout their career. Number of events experienced ranged from one to more than 10. | Approximately one-fifth of midwives were experiencing posttraumatic stress symptoms at clinically significant levels. Higher resilience and trait emotional intelligence scores were associated with reduced posttraumatic stress symptoms. Higher empathy, perceived social support, and resilience were associated with higher trait emotional intelligence. Lower resilience significantly predicted posttraumatic stress symptoms. | Eighteen (16%) changed their professional allocation after experiencing a traumatic perinatal event, and 26 (24%) had considered doing so. Thirteen midwives had taken sick leave following exposure to a traumatic perinatal event (12%) and 27 had considered doing so (25%). Forty-one midwives had seriously considered leaving the profession following exposure to a traumatic perinatal event (37%). | Not mentioned |  |  |
|  | Nuzum D (2014) | obstetricians | Stillbirth | The human response to stillbirth was characterised by the personal impact of stillbirth for consultants and, in turn, how that shapes the care they provide. The depth and complexity of the personal impact of stillbirth revealed feelings of loss, fear, remorse, sadness, discomfort and anger. The weight of professional responsibility was characterised by the sense of professional burden and the possibility of a medico-legal challenge—mostly for those who are primarily gynaecologists resulting in the question ‘what have I missed?’. | Not mentioned | This feeling of collegiality or lack of it was also conveyed by a competitive undercurrent expressed by some consultants about how stillbirth care should be managed. |  |  |
|  | Oe M (2018) | Midwives | Not indicated | The mean total STSS score was significantly higher in the Swiss sample (31.8 ± 9.7) compared with the Japanese sample (24.1 ± 8.6; \|z\| = 4.56, p < 0.01). | Not mentioned | Not mentioned |  |  |
|  | Pastor Montero (2011) | Nurses, midwives, nursing auxiliaries, obstetricians | Perinatal loss | The professionals manifest feelings of sorrow, anxiety, insecurity, resentment, guilt, rage, feeling of failure and impotence, which are mainly related with not knowing how to face and manage these situations. | Frequently, the emotional response is to avoid the situation in order to control professional stress. The consulted bibliography reveals that perinatal loss exerts a strong emotional impact not only on the parents and their environment, but also on the care professionals (4). | Not mentioned |  | The conclusion  reached is that the promotion of training programs to acquire knowledge, skills and abilities in management of perinatal bereavement and the development of a clinical practice guideline for perinatal loss are necessary. |
|  | Rice H (2013) | Midwives | Traumatic stress from witnessing and working with (potentially) traumatised clients.  Feeling ‘stuck’ between wishing they could practice according to their midwifery philosophy and the reality of working within a medical model of care. | Feelings of responsibility for women and babies’ outcomes, and repeatedly questioning what they could have done differently to prevent a traumatic birth was also reported. Feeling for the woman emerged as a major factor in midwives’ experiences of witnessing traumatic birth. | Not mentioned | Participant felt that there was little support for midwives within the hospital system to debrief their experiences and she felt her feelings were overlooked and ignored by her workplace. |  |  |
|  | Robinson K (2022) | Registered nurses, nurse-midwives (CNM), attending physicians and resident physicians | Traumatic Childbirth Events TCE were defined as events that may provoke psychological distress and defensive practice behaviors, specifically, shoulder dystocia, postpartum hemorrhage, stillbirth/infant death, unsuccessful newborn resuscitation, maternal death, uterine rupture, and instrument injury to mother or newborn. | CNM participants reported that TCE exposure had a strong effect on their professional practice in comparison to the effect experienced by attending and resident physicians. RNs described a strong desire to leave the maternity unit and pursue careers in other areas of nursing. | Not mentioned | Not mentioned |  |  |
|  | Schrøder K (2016a)  Response rate was 59% of which 85% (n=1027) stated that they had been involved in a traumatic childbirth. | Obstetricians and midwives | Traumatic childbirth was defined as a birth where the infant or mother had suffered presumed permanent, severe and possibly fatal injuries related to the birth. | Midwives reported significantly higher scores on the Copenhagen Psychosocial Questionnaire than obstetricians, to a minor extent during the most recent four weeks and to a greater extent immediately following a traumatic childbirth scale, indicating higher levels of self-reported psychosocial health problems.  Sub-group analyses showed that this difference might be gender related. | At the time of the survey, 21% no longer worked on the labour ward. Of these, 25% responded that they had left primarily of partly because they felt the responsibility was too great a burden to carry. | Not mentioned |  |  |
|  | Schrøder K (2016b) | Obstetricians and midwives | Traumatic childbirth was defined as a birth where the infant or mother had suffered presumed permanent, severe and possibly fatal injuries related to the birth. | The inner struggles with issues of blame, guilt and existential considerations were dominant. Feelings of guilt were reported by 36–49%, and 50% agreed that the traumatic childbirth had made them think more about the meaning of life.  When asked what impact the traumatic birth had had on their lives, some of the participants of the interview study had considered whether they would be capable of going through a similar ordeal in the future. | Sixty-five percent felt that they had become a better midwife or doctor due to the traumatic incident.  Some participants had briefly considered leaving their profession. | A total of 30% worried to a great or to some extent about what their peers would think of them after the event, 34% to a small extent, and 35% not at all. “Twelve percent strongly agreed or agreed with  “Comments or behavior from one or several colleagues caused more guilty feelings and/or lower self-esteem” and 87% disagreed or strongly disagreed. |  |  |
|  | Schrøder K (2019) | Obstetricians and midwives | Traumatic childbirth was defined as a birth where the infant or mother had suffered presumed permanent, severe and possibly fatal injuries related to the birth. |  | Significantly more midwives (32%) than obstetricians (21%) found it difficult to ‘a great’ or to ‘some extent,’ to continue clinical  practice after the event, and that to ‘a great’ or to ‘some extent,’  there were times when they felt less able to work safely and effectively because of what had happened (midwives: 35%; obstetricians:  29%; P = <0.05).  Both midwives (32%) and obstetricians (32%) feared having to speak to the patient and/ or family after the event. | Finally, we asked the respondents whom they talked to about the event (Fig. 1). The three most frequent categories of choice were colleagues on the same ward (n = 561; 95%), partner (n = 403; 68%) and management (n = 292; 49%). Management and family were chosen more frequently by midwives than doctors (52% vs.  38% and 33% vs. 23%, respectively) when considering who to talk to about the event (P= < 0.05).  Respondents experienced high levels of social support from colleagues and social community at work, midwives significantly higher than obstetricians, and 95% talked to colleagues about an adverse event. |  |  |
|  | Sheen K (2015)  16% response rate of which 95% witnessed and 76% listened to an account from a woman in their care about a traumatic perinatal event. | Midwives | Event where responders perceived that the mother/child were at serious risk (dying/serious injury) and that the midwife experienced a sense of fear, helplessness or horror.  They could be part of it but also listened to an account from a woman in their care. | 32% exceeded the cut of score indicative of symptoms corresponding with a clinical diagnosis of PTS.  Empathy and previous trauma exposure (personal and whilst providing care to women) were associated with more severe posttraumatic stress responses. Symptoms of posttraumatic stress were associated with negative worldview beliefs and two domains of burnout. | Taken time of sick: 12%  Professional allocation  - Short-term: 20%  - Long-term 14% . Seriously considered leaving (35%) | Not mentioned | Symptoms of PTS were associated with elevated symptoms of burnout, namely emotional exhaustion and reflected the depersonalisation of recipients of care.  Higher empathy and personal trauma history were associated with higher symptoms of posttraumatic  stress, but their predictive utility was limited.  Symptoms of posttraumatic stress disorder were associated with elevated levels of burnout and held implications for midwives’ decisions to remain within the profession. |  |
|  | Sheen K (2016a) | Midwives | Unexpected episodes contributing to feeling ‘out of a comfort zone’. Sudden, unpredictable and uncontrollable events. Some events included situations where access to personnel or resources was limited or delayed. For example, when waiting a long time for theatre staff to  arrive;  Midwives perceived events to be more difficult when they held an existing relationship with the mother. | Midwives with high distress following a traumatic perinatal event were more likely to feel personally upset and perceive all aspects of their life (personal and professional) to be adversely affected.  1. Emotionally distraught; feelings of shock and despair  2. Self-blame and guilt feelings of vulnerability and judgement  3. Attempting to make sense of what happened  4. Feelings of vulnerability and judgement  5. A permeating impact on professional life  6. An enduring psychological impact  Midwives reported changes in the way they felt and general demeanour following the event. This ranged in severity; some felt low in their mood, others report serious implications for their psychological health. Family and home lives were also impacted.  Midwives with high distress reported impacts to their personal lives; for example, becoming fearful about adverse events occurring to other people in their life or vigilant for the safety of those around them. | Midwives reported practising in an increasingly defensive manner to prevent similar occurrences happening again, or felt less confident in their practice.  Midwives valued the opportunity to learn from their experience and improve their future practice. This included practical changes to procedure or protocol in organisational settings and personal changes to practice (e.g., becoming more assertive).  Midwives also reported changing clinical allocation or considering leaving the profession altogether. | Wanting to talk about it: accessing and receiving helpful support from peers.  Being reassured (by colleagues).  Gaining an objective perspective by talking through the event  Not feeling acknowledged by senior colleagues.  Having to seek own (professional) help.  Midwives with high levels of distress reported stress specifically in relation to a perceived lack of staff, low morale in the workplace, and limited resources in their job role.  Some midwives perceived contact from senior colleagues, managers or supervisors of midwives to be lacking, difficult to access, or (when received) to have a punitive focus. | Both groups reported ‘going into auto-pilot’, ignoring their feelings and focusing on completing the relevant procedures to maintain a professional appearance.  Midwives did not always feel prepared to experience trauma, or supported in their workplace after a traumatic perinatal event. |  |
|  | Sheen K (2016b) | Midwives | Themes indicated that events were characterised as severe, unexpected and complex. They involved aspects relating to the organisational context; typically limited or delayed access to resources or personnel.  Sometimes, but not always, involving adverse or enduring implications.  Difficult accessing resources or personnel  Ante partum haemorrhage at 42weeks, transferred her to theatre for LSCS, was unable to get an anaesthetist for 30minutes.[ID 203 W]  Massive- PPH at home- woman on own with baby – felt her life ebbing away whilst waiting for ambulance.[ID372H]  (2) The mother was alone Maternal collapse due to PPH following delivery. Woman  wasn't 'seen to' for 5 minutes unable to reach call bell and on own in room.[ID19H]  Both being part of the event and being “outside” could be traumatic. If a midwife listened to the event, witnessing parents’ distress was hard. | Traumatic events had a common theme of generating feelings of responsibility and blame  Personal salience resonated in some way with the midwife’s own life experience. | Not mentioned | Lack of support from other colleagues (after the event).  Difficult to access help under the event. | Midwives with high distress had a greater propensity to perceive all aspects of personal and professional lives to be adversely impacted, and reported more difficulty in accessing support from peers and senior colleagues.  Midwives are exposed to events as part of their work that they may find traumatic. Understanding the characteristics of the events that may trigger this perception may facilitate prevention of any associated distress and inform the development of supportive interventions. |  |
|  | Sheen K (2022)  The initial response rate to the survey was low at 17% N=1095 | Obstetricians and gynaecologists | Events perceived as traumatic  by obstetricians and gynaecologists | 728 (66%) reported work-related trauma experience, with  525 providing a brief description of an event.  Events perceived as traumatic were  maternal or neonatal death/stillbirth,  haemorrhage and events involving a difficult delivery | Not mentioned | An absence of support was reported by participants. Furthermore, feeling criticised, gossiped about or blamed by other members of the team were also reported. |  |  |
|  | Slade P (2018)  No response rate given. The majority of participants (n = 136, 89%) reported that they had experienced a work-related traumatic event at some point during their career as a midwife. Over twothirds (n = 104, 68%) reported that they had experienced a traumatic perinatal event while working as a midwife in the past 5 years. | Midwives | The definition of a traumatic perinatal event was provided in the questionnaire, and corresponded to Criterion A of the DSM-IV for PTSD (APA 2010). | Fourteen per cent reported symptoms of PTSD commensurate with a clinical diagnosis (n = 19) and an additional 10% reported subclinical levels.  Just under 40% (n = 59, 39%) reported high emotional exhaustion, 7% (n = 10) reported high levels of depersonalization and 28% (n = 41) reported low levels of personal  accomplishment within their professional role. | Over one-third of participants had seriously considered leaving their current organization (n = 54/150, 36%), and a similar proportion had seriously considered leaving midwifery (n = 53/150, 35%).  Almost one-quarter of midwives reported that, in the past 6 months, they had taken time off work owing to stress as a result of trauma exposure (n = 36/150, 24%), 7% reported that they had changed or had seriously considered changing their clinical allocation on a short-term basis (e.g. one or two shifts), and 10% had changed or seriously considered changing their clinical allocation on a long-term basis. | Not mentioned |  |  |
|  | Slade P (2020)  Response rate was 18% (n=1095). Two-thirds reported exposure to traumatic work-related events. | Obstetricians and gynaecologists | An event involving actual (or threatened) death or serious injury. | 18% reported clinically significant PTSD symptoms. Staff of black or minority ethnicity were at increased risk of PTSD. Clinically significant PTSD symptoms were associated with lower job satisfaction, emotional exhaustion and depersonalisation.  The impacts of traumatic events were experienced both immediately and in the longer term. | Organisational impacts included sick leave, and ‘seriously considering leaving the profession’.  They affected all aspects of personal and professional lives leading to high  levels of anxiety around particular procedures or more generally in the workplace and in some cases a distancing from engagement with patients. | 91% wanted a system of care with specific support in relation to trauma responses provided. The culture in obstetrics and gynaecology was identified as a barrier to trauma support.  What helped in managing the impact of traumatic  events was focused on sharing of the experience with a supportive  team and support from seniors preventing a sense of  isolation. In particular, the No PTSD group reported help from receiving support and time to process the event. For  both PTSD and No PTSD groups, the role of support from  family/friends and external input was noted. | Exposure to work-related trauma is a feature of the experience of obstetricians and gynaecologists. Some will suffer PTSD with high personal, professional and organisational impacts. A system of care is needed. |  |
|  | Toohill (2019)  84% response rate in a convenient sample. 85.4% indicated they had experienced birth-related trauma in their professional role (n = 199). | Midwives | Either personally and /or professionally experienced birth as traumatic. Witnessing the disrespect of women. Being disrespected as a midwife.  Being complicit in poor care practices.  Working with, and living in, the reality that not everything goes according to plan. Situational and workplace factors intensifying stress, such as unexpected situations, staff conflict, understaffing, culture of blame, censure by other professionals, fear of litigation. | There was no statistical relationship between previous trauma (personal or professional) and scores on the Practice Concern Scale.  Stress and anxiety escalated in situations where midwives felt  unsupported and lacked the resources they needed to provide  appropriate care to women and their families. | Not mentioned | Not mentioned |  |  |
|  | Wahlberg Å (2017a)  The response rate was 39.9% (n = 1459) for midwives and 47.1% (n = 706) for obstetricians. 84% of the obstetricians and almost 71% of responding midwives had experienced one or more self-reported severe obstetric event with detrimental consequences for the woman or the newborn. | Midwives and obstetricians | Traumatic event were defined as intrapartum death; severe asphyxia at birth; severe injury at delivery; neonatal death from delivery-related causes; maternal death; very severe or life-threatening maternal morbidity; and other stressful events during delivery, such as exposure to aggression or violence by the patient or by a family member of the patient in conjunction with a delivery.  Of the staff who had experienced a severe event, 10.3% of midwives (n = 107) and 22.1% of the obstetricians (n = 131) reported negative reactions from the patient or the patient’s family after the event.  Almost 15% of the midwives and 18% of the obstetricians reported other stressful events such as violence or aggression. The related comments most frequently referred to aggressive or threatening partners, sometimes affected by drugs or alcohol. 14% of the midwives and 22.4% of the obstetricians reported that they had faced complaints of medical negligence from the patient or the family of the patient, after the worst severe event. | Not mentioned | Not mentioned | Not mentioned |  |  |
|  | Wahlberg Å (2017b) | Midwives and obstetricians | Traumatic event  were defined such as (1) the child died during birth or had severe asphyxia, neonatal death due to asphyxia or other birth-related injury; (2) maternal near-miss, maternal death during delivery; and  (3) other difficult and threatening events during obstetric care, as defined by the respondents, for example violence or threat. | 43% reported emotions of intense fear, helplessness or panic in connection with the severe event.  Emotions of guilt were experienced by 47% of the obstetricians and 28% of the midwives after the traumatic experience.  Seventy percent of the obstetricians and 66% of the midwives had at least one symptom of re-experiencing the event and 40% of the obstetricians and 35% of the midwives had at least one symptom of avoidance or numbing after the event.  In all, 15% reported partial PTSD or PTSD, while all the symptom criteria for ‘full’ PTSD were fulfilled by 7% of the obstetricians and 5% of the midwives.  For both obstetricians and midwives, emotions of guilt, negative reactions from parents, the experience of insufficient support from local managerial staff, colleagues, friends and partner as well as bad experiences during reassembly (debriefing) are significant factors in the development of PTSD/partial PTSD. | A threat to their professional role/identity in the moment of exposure was reported by 25% of the obstetricians and 17% of the midwives.  The obstetricians reporting partial or probable PTSD more often changed their job situation by stopping working on the delivery unit, stopping being on call or changing their role to outpatient care.  Midwives who reported symptoms of partial or probable PTSD following exposure to a severe event reported significantly higher levels of sick leave than obstetricians did. Healthcare providers with symptoms of partial PTSD or probable PTSD in both groups changed their work to a significantly higher degree. | 45% of the exposed obstetricians had participated in a reassembly (debriefing) after the event, of whom 27% exposure to an event that was perceived as the worst during their working life on the labour ward.  Participants indicate insufficient support from local managerial staff, colleagues, friends and partner. |  | For developing partial  PTSD/ PTSD remained statistically significant after  adjusting for emotions of guilt and insufficient support from  friends for both obstetricians and midwives, while negative  reactions from parents remained significant only for obstetricians,  and experiencing insufficient support from colleagues  remained significant only for midwives. |
|  | Wahlberg Å (2019) | Midwives and obstetricians | Severe medical events in which patients are badly hurt. In birth care, escalating situations can result in death or injury to a mother or newborn child (‘second victim’). | The obstetricians and midwives described how they struggled with a torrent of conflicting emotions of guilt and shame. Guilt for what had been done, for the suffering and pain inflicted on the woman and the child, and shame for not being a better, more insightful and knowledgeable professional. | Other midwives and obstetricians were finding the price too high and their experiences of the severe events and the aftermath had resulted in more drastic changes in their work-related worldview. They described that they had lost their naivety, security or faith in “the system”, represented by women, colleagues, managers, the medico-legal system and the media.  The participants described how their experiences of the severe event and the process afterwards had made them reflect and also make some work-related changes.  There were also those who, when scrutinizing how the present working situation affected them, started to think about or actively search for new working environments. | Sometimes emotional reactions could partly be compensated for them by being provided close support from insightful colleagues who would listen and validate the experiences.  Preventive actions taken after an event were sometimes considered window-dressing, creating imaginary safety.  There were also participants for whom the event had entailed proof to themselves that they could live through a work-related crisis and that people had stood up for them. This feeling of continuously being valued and supported at their work places made it possible for them to continue, making some minor changes in work duties. | Based on the experience of outer and inner factors, the outcome was interpreted as varying between fully regaining or even further developing the professional identity based on experiences of having gone through professional hardships, with backing and support. It could also imply leaving birth care because the price was too high. Leaving birth care or work altogether was sometimes out of necessity rather than by choice. Reconsidering future work was another possible outcome. |  |
|  | Wahlberg Å (2020) | Midwives, and obstetricians | Events during childbirth when the child died or was severely injured; maternal near-miss; maternal mortality; and other events such as violence or threat. | Our findings enhance an understanding of the complexity of labour care and the heavy emotional toll that an injured or deceased child or mother can have on the healthcare professionals involved.  When the severity of the situation became clear, “cognitive and emotional discordance was experienced”, and, in the aftermath, the professionals described a “search for internal and external redemption” related to strong emotions of being left alone. A main theme was that midwives and physicians felt left alone with the emotional surge.  The informants described having strong emotional reactions during the severe events, a horror of losing control, as well as feelings of guilt, blame and shame in the aftermath when internal as well as external redemption was sought. | Not mentioned | A common description was the limited support given after a severe event. Midwives described how they had received appreciated patronage  from their “colleagues on the floor” early on in their professional career, but how a tougher working climate and related time constraints no longer allowed for the time and flexibility necessary for such reassurance. Professionals had been offered the option of talking to occupational healthcare representatives (counsellors or psychologists employed to assist the hospital workforce), which was, with few exceptions, not appreciated. |  |  |
|  | Wallbank S (2013)  54% of eligible staff responded. | Doctors, midwives and nurses | Perinatal death | Impact of Events Scale (IES) scores revealed 55% of participants reporting subjective distress levels indicating a ‘high’ level of clinical concern. | Not mentioned | Staff perceptions of support outside work significantly predicted distress (p = 0.023; CI -4.818 to -0.355). Working environment, specifically lack of supervisor support, was significantly correlated with negative coping strategies (r = -0.242, p = 0.001). |  |  |
|  | Walker AL (2020)  Response rate was 30.2%. Nearly all (n=31, 96.9%) had been exposed to some sort of traumatic birth. Nearly 72% (n = 23) of respondents had experienced at least one adverse professional event (claim, etc.), with over 95% (n = 22) finding their experiences traumatic. | Obstetricians | Unexpected intrapartum stillbirth, unexpected neonatal death, maternal death or near miss, other traumatic birth or intrapartum emergency, other work-related trauma during birth such as violence/threat to self. Professional event, such as personal criticism in morbidity and mortality meeting, incident report made about your management, formal patient complaint, performance review made by your employer after an adverse event, named in a medicolegal claim, media coverage critical of your management, reported to medical board or other regulatory authority, restrictions on or suspension from practice. | Three‐quarters had current symptoms of traumatic stress; one‐quarter had symptoms of work‐related burnout, but over two‐fifths reported significant post‐traumatic growth. | One quarter (n = 8) of respondents had considered leaving obstetrics due to their experience, but were still practising. | There is a ‘culture of blame in obstetrics’, and only ‘in some workplaces it’s supportive and safe’.  Only eight (25%) respondents felt that their employer provided sufficient support after the adverse event. Half (n = 16) reported receiving sufficient support from family. Of the half (n = 16) who accessed professional counsellors, 75% (n = 12) found this ‘not at all’ supportive. |  |  |
